# Integrated Bayesian analysis of rare exonic variants to identify risk genes for schizophrenia and neurodevelopmental disorders

**DOI:** 10.1186/s13073-017-0497-y

**Published:** 2017-12-20

**Authors:** Hoang T. Nguyen, Julien Bryois, April Kim, Amanda Dobbyn, Laura M. Huckins, Ana B. Munoz-Manchado, Douglas M. Ruderfer, Giulio Genovese, Menachem Fromer, Xinyi Xu, Dalila Pinto, Sten Linnarsson, Matthijs Verhage, August B. Smit, Jens Hjerling-Leffler, Joseph D. Buxbaum, Christina Hultman, Pamela Sklar, Shaun M. Purcell, Kasper Lage, Xin He, Patrick F. Sullivan, Eli A. Stahl

**Affiliations:** 1 Division of Psychiatric Genomics, Department of Genetics and Genomic Sciences, Institute for Genomics and Multiscale Biology, Icahn School of Medicine at Mount Sinai, New York, 10029 NY USA; 2 Department of Medical Epidemiology and Biostatistics, Karolinska Institutet, Stockholm, Sweden; 3 Stanley Center for Psychiatric Research, Broad Institute of MIT and Harvard, Cambridge, Massachusetts USA; 4 Department of Surgery, Massachusetts General Hospital, Boston, 02114 MA USA; 5 Charles Bronfman Institute for Personalized Medicine, Icahn School of Medicine at Mount Sinai, New York, 10029 NY USA; 6 Laboratory of Molecular Neurobiology, Department of Medical Biochemistry and Biophysics, Karolinska Institutet, Stockholm, SE-17177 Sweden; 7 Division of Genetic Medicine, Departments of Medicine, Psychiatry and Biomedical Informatics, Vanderbilt Genetics Institute, Vanderbilt University Medical Center, Nashville, 37235 TN USA; 8 Department of Genetics, Harvard Medical School, Cambridge, Massachusetts USA; 9 Verily Life Sciences, 269 E Grand Ave, South San Francisco, 94080 CA USA; 10 Seaver Autism Center, Department of Psychiatry, Icahn School of Medicine at Mount Sinai, New York, 10029 NY USA; 11 The Mindich Child Health and Development Institute, Icahn School of Medicine at Mount Sinai, New York, 10029 NY USA; 12 Friedman Brain Institute, Icahn School of Medicine at Mount Sinai, New York, 10029 NY USA; 13 Department of Functional Genomics, The Center for Neurogenomics and Cognitive Research, VU University and VU Medical Center, Amsterdam, The Netherlands; 14 Department of Molecular and Cellular Neurobiology, The Center for Neurogenomics and Cognitive Research, VU University, Amsterdam, The Netherlands; 15 Sleep Center, Brigham and Women’s Hospital, Harvard Medical School, Boston, Massachusetts USA; 16 Department of Human Genetics, University of Chicago, Chicago, 60637 IL USA; 17 Departments of Genetics and Psychiatry, University of North Carolina, Chapel Hill, 27599-7264 North Carolina USA

**Keywords:** De novo mutations, Rare variants, Schizophrenia, Autism, Developmental disorders, Intellectual disability, Epilepsy, Hierarchical model

## Abstract

**Background:**

Integrating rare variation from trio family and case–control studies has successfully implicated specific genes contributing to risk of neurodevelopmental disorders (NDDs) including autism spectrum disorders (ASD), intellectual disability (ID), developmental disorders (DDs), and epilepsy (EPI). For schizophrenia (SCZ), however, while sets of genes have been implicated through the study of rare variation, only two risk genes have been identified.

**Methods:**

We used hierarchical Bayesian modeling of rare-variant genetic architecture to estimate mean effect sizes and risk-gene proportions, analyzing the largest available collection of whole exome sequence data for SCZ (1,077 trios, 6,699 cases, and 13,028 controls), and data for four NDDs (ASD, ID, DD, and EPI; total 10,792 trios, and 4,058 cases and controls).

**Results:**

For SCZ, we estimate there are 1,551 risk genes. There are more risk genes and they have weaker effects than for NDDs. We provide power analyses to predict the number of risk-gene discoveries as more data become available. We confirm and augment prior risk gene and gene set enrichment results for SCZ and NDDs. In particular, we detected 98 new DD risk genes at FDR < 0.05. Correlations of risk-gene posterior probabilities are high across four NDDs (*ρ*>0.55), but low between SCZ and the NDDs (*ρ*<0.3). An in-depth analysis of 288 NDD genes shows there is highly significant protein–protein interaction (PPI) network connectivity, and functionally distinct PPI subnetworks based on pathway enrichment, single-cell RNA-seq cell types, and multi-region developmental brain RNA-seq.

**Conclusions:**

We have extended a pipeline used in ASD studies and applied it to infer rare genetic parameters for SCZ and four NDDs (https://github.com/hoangtn/extTADA). We find many new DD risk genes, supported by gene set enrichment and PPI network connectivity analyses. We find greater similarity among NDDs than between NDDs and SCZ. NDD gene subnetworks are implicated in postnatally expressed presynaptic and postsynaptic genes, and for transcriptional and post-transcriptional gene regulation in prenatal neural progenitor and stem cells.

**Electronic supplementary material:**

The online version of this article (doi:10.1186/s13073-017-0497-y) contains supplementary material, which is available to authorized users.

## Background

Integrating rare variation from family and case–control (CC) studies has successfully implicated specific genes contributing to risk of neurodevelopmental disorders (NDDs) including autism spectrum disorders (ASD), intellectual disability (ID), developmental disorders (DDs), and epilepsy (EPI). These early-onset disorders typically manifest as infant or childhood developmental delay or regression, and can be co-morbid even within individuals [1] at the symptom and syndrome levels. ASD typically includes deficits in social function and often includes cognitive deficits. ID is defined by severe cognitive deficits. DD is characterized by physical or neurological developmental delays frequently including ID while EPI is defined by recurrent seizures and often occurs in probands of the other NDDs [2–4]. Cognitive dysfunction is a common thread among these disorders and many of the risk genes identified for them point to brain neuronal development as well as synaptic function.

For schizophrenia (SCZ), however, while sets of genes have been implicated through studying rare variation (including NDD risk genes) [5–7], only two risk genes containing rare exonic variants with a strong effect have been identified [6, 8, 9]. SCZ is an etiologically complex psychiatric disorder characterized by hallucinations, delusions, and cognitive symptoms. Heritability is estimated to be 60–80 *%* [10, 11] and the genetic architecture of SCZ is highly polygenic with contributions from common variation and rare inherited and de novo (DN) structural and exonic variants [5–8, 12–15]. With the advent of affordable high-quality next-generation sequencing, the genetics of SCZ and other diseases are increasingly being better characterized, especially for rare variants. Rare variants in CC and trio samples have been leveraged to identify SCZ genes and gene sets. However, the SCZ rare-variant genetic architecture remains poorly understood. Such analyses could help gain further insights into this disease, for example, by using the estimated number of risk genes to calibrate false discovery rates (FDRs) for gene discovery or by using the distribution of effect sizes to improve power estimates and rare-variant association study design. A better understanding of our certainty for sets of risk genes for SCZ will provide a better picture of biological pathways relevant for the disease.

We developed an improved hierarchical Bayesian modeling framework [16], Extended Transmission and de novo Association (extTADA), to analyze whole exome sequence data in SCZ and four NDDs (ASD, ID, DD, and EPI), which have substantial clinical and etiological overlap. All are brain diseases with prominent impacts on cognitive function. Multiple recent studies supporting genetic overlap among these disorders have included common variant genetic correlations [17, 18], shared molecular pathways [19, 20], and shared genes with DN mutations [6, 21]. Using the largest sample assembled to date for a unified analysis of these disorders, we find greater overlap among the NDDs than with SCZ, despite the emphasis on overlap in the SCZ rare-variant literature [6, 7, 19]. We used the statistical support of extTADA to compile a comprehensive list of 288 NDD genes. Network analyses of these genes are beginning to pinpoint and intersect functional processes implicated in disease, brain cell types, and developmental time points of expression.

## Methods

### Data

Additional file 1: Figure S1 shows the workflow for all data used in this study.

#### Variant data for SCZ, ID, DD, EPI, and ASD

High-quality variants were obtained from published analyses as shown in Additional file 1: Table S1. These included DN data for SCZ and four NDDs, and CC data for SCZ and ASD. Quality control and validation for these data were carried out within the original studies (Additional file 1: Table S1). To maintain consistency across data sets, we re-annotated all of the variants in our analyses. For SCZ CC data, we performed exome-wide association analyses with and without covariates to test for stratification, and used clustering of CC samples to identify non-heterogeneous samples for extTADA analysis (see Additional file 1: Methods).

Variants were annotated using Plink/Seq (using RefSeq gene transcripts and the UCSC Genome Browser [22]) as described in Fromer et al. [6]. SnpSift version 4.2 [23] was used to annotate these variants further using dbnsfp31a [24]. Variants were annotated as follows: loss of function (LoF) (nonsense, essential splice, and frameshift variants); missense damaging (MiD) (defined as missense by Plink/Seq and damaging by each of seven methods [7]: SIFT, Polyphen2_HDIV, Polyphen2_HVAR, LRT, PROVEAN, MutationTaster, and MutationAssessor); missense; synonymous mutations within DNase I hypersensitive sites (DHSs) [25], using http://wgEncodeOpenChromDnaseCerebrumfrontalocPk.narrowPeak.gz from ENCODE [26, 27] (downloaded 20 April 2016); and synonymous. Based on previous results with SCZ exomes [5, 7], only CC singleton variants were used in this study (i.e., they were observed once). The data from the Exome Aggregation Consortium (ExAC) [28] were used to annotate variants as inside ExAC (InExAC or not private) or not inside ExAC (NoExAC or private), using ExAC.r0.3.nonpsych.sites.vcf.gz (downloaded from [29] 20 April 2016) and BEDTools.

The variant categories used in extTADA were LoF, MiD, and silent within frontal cortex-derived DHS peaks (silentFCPk).

#### Mutation rates

We used the methodology based on trinucleotide context [30, 31] and incorporating depth of coverage [6] to obtain mutation rates for each variant annotation category. We assigned 1/10 of the minimum non-zero mutation rate to genes with calculated mutation rates equal to zero.

#### Gene sets

Multiple resources were used to obtain gene sets for our study. First, we used known and candidate gene sets with prior evidence of involvement in SCZ and ASD. Second, to identify possible novel significant gene sets, we collected genes sets from available data bases (see below).

**Known/candidate gene sets**

These gene sets and their abbreviations are presented in Additional file 1: Table S2. They included: gene sets enriched for ultra rare variants in SCZ which were described in detailed in Supplementary Table 5 of [7] consisting of missense constrained genes (constrained) from [30], loss-of-function intolerant genes (pLI90) from [28], *RBFOX2* and *RBFOX1/3* target genes (rbfox2, rbfox13) from [32], Fragile X mental retardation protein target genes (fmrp) from [33], *CELF4* target genes (celf4) from [34], synaptic genes (synaptome) from [35], microRNA-137 (mir137) from [36], PSD-95 complex genes (psd95) from [37], ARC and NMDA receptor complexes (arc, nmdar) genes from [38], and *de novo* copy number variants in SCZ, ASD and bipolar disorder; allelic-biased expression genes in neurons from Table S3 of [39]; promoter targets of *CHD8* from [40]; known ID gene set from the Sup Table 4 and the 10 novel genes reported by [41]; gene sets from MiD and LoF *de novo* mutations of ASD, EPI, DD, ID; the essential gene set from the supplementary data set 2 of [42]; lists of human accelerated regions (HARs) and primate accelerated regions (PARs) [43] (downloaded May 11, 2016 from [44]; genes within 100kb [45]) (geneInHARs, geneInPARs); known epilepsy genes from Supplementary Table 3 of [46]; common-variant genes from Extended Table 9 of [15]; 24 co-expression modules from Supplementary Table 2 of [47]; and 134 gene sets from mouse mutants with central nervous system (CNS) phenotypes were obtained from [15, 48].

In the gene-set tests for a given disease, we removed the list of known genes and the list of DN mutation genes for that disease. As a result, we tested 185 candidate gene sets for ASD, DD, and SCZ, and 184 candidate gene sets for EPI and ID.

**Other gene sets**

We also used multiple data sets to identify novel gene sets overlapping with the current gene sets. We assembled gene sets from the Gene Ontology data base [49], KEGG, and REACTOME, and the C3 motif gene sets collected for the Molecular Signatures Database (MSigDB) [50] plus the gene sets from The Mouse Genome Database [51]. To increase the power of this process, we used only gene sets with between 100 to 4,995 genes. In total, there were 2,084 gene sets. These gene sets and the above gene sets were used in this approach.

#### Transcriptomic data

Spatiotemporal transcriptomic data were obtained from BRAINSPAN [52]. The data were divided into eight developmental time points (four prenatal and four postnatal) [53]. Single-cell RNA-seq data were obtained from [54].

### The extTADA pipeline

Recently, He et al. developed the Transmission and de novo Association (TADA) pipeline, which integrates DN and inherited (or CC) variants to increase power in the identification of risk genes for ASD [16, 31]. TADA borrows information across variant categories of DN and CC samples in gene-level association analysis, which is critical for sparse rare-variant sequence data, and showed better power than the traditional approach of combining *p* values from multiple data sets using Fisher’s method [16].

TADA assumes that a proportion of all genes (*π*) comprise risk genes. Therefore, for each gene, TADA compares two hypotheses: risk gene (*H*_1_) or non-risk gene (*H*_0_). The method combines multiple categories of DN and CC variants; however, TADA is an empirical Bayesian association method with respect to model parameters and does not provide any uncertainty information (e.g., confidence intervals) [16]. TADA uses a simple CC model with parameter ranges that can imply protective variants in its CC model [16, 31]. Here, we extend TADA into a flexible and convenient model, which can be applied to different population samples, including DN and CC data alone or in combination. The new pipeline, Extended Transmission and de novo Association, extTADA (Additional file 1: Figure S2 and Table S3), uses a Markov chain Monte Carlo (MCMC) approach to sample the joint posterior of all genetic parameters given all variant categories, in one step. The current pipeline provides Bayesian credible intervals (CIs) for estimated parameters.

Additional details are in Additional file 1: Methods and https://github.com/hoangtn/extTADA. Briefly, for a given gene, all variants of a given category (e.g., either DN or singleton CC LoF) were collapsed and considered as a single count. Let *γ* be the relative risk (RR) of the variants, which is assumed to follow a distribution across risk genes: $\gamma \sim \text {Gamma}(\bar {\gamma } \times \beta, \beta)$. $\bar {\gamma }$ and *β* are hyperparameters of *γ* as presented in Additional file 1: Table S3. The data likelihood was considered a mixture of non-risk and risk-gene hypotheses, *H*_0_: *γ*=1 and *H*_1_: *γ*≠1:

1 $$  P(x|H_{1}, H_{0}) = \pi P(x | H_{1}) + (1 - \pi) P(x | H_{0}),  $$

where *H*_0_ and *H*_1_ represent *γ* and all other parameters under the model, and the mixture proportion *π* is interpreted as the proportion of risk genes genome-wide.

The data *x* are DN and CC variant counts (*x*_*dn*_,*x*_*ca*_,*x*_*cn*_ for de novo, case and control data respectively). We assumed that these data are from independent variant categories and independent population samples. The extTADA likelihood is the product of data probabilities over any number of population samples and variant categories. The hyperparameters ($\bar {\gamma }$ and *β*) for different categories and *π* (Additional file 1: Table S3) were jointly estimated based on the mixture model,

2 $$  P(x|\phi_{1}, \phi_{0}) = \prod \limits_{i=1}^{\text{Gene Number}} \left[ \pi P_{1i} + (1 - \pi) P_{0i} \right],  $$

where *ϕ*_1_ and *ϕ*_0_ are sets of parameters of all population samples and categories. *P*_1*i*_ and *P*_0*i*_ at the *i*th gene were calculated across population samples and categories as follows:

$$ \begin{aligned} P_{ji} & = P_{ji}(x_{i}|\phi_{j}) \\ & = \left[ P_{ji(\text{dn})}(x_{i(\text{dn})}|\phi_{j(\text{dn})}) \right] \left[ P_{ji(\text{cc})}(x_{_{i}(\text{ca})}, x_{i(\text{cn})}|\phi_{j(\text{cc})}) \right] \\ & = \left(\prod \limits_{h=1}^{N\text{dn}_{\text{pop}}} \prod \limits_{k=1}^{C\text{dn}} P_{ji(\text{dn})_{hk}}(x_{i(\text{dn})_{hk}}|\phi_{j(\text{dn})_{hk}}) \right) \\ & \quad \times \left(\prod \limits_{a=1}^{N\text{cc}_{\text{pop}}} \prod \limits_{b=1}^{C\text{cc}} P_{ji(\text{cc})_{\text{ab}}}(x_{i(\text{ca})_{\text{ab}}},x_{i(\text{cn})_{\text{ab}}}|\phi_{j(\text{cc})_{\text{ab}}}) \right), \quad j = 0, 1. \end{aligned} $$

*N*dn_pop_ and *N*cc_pop_ are the numbers of DN and CC population samples, and *C*_dn_ and *C*_cc_ are the numbers of annotation categories in the DN and CC data.

To simplify the estimation process in Eq. 2, we approximated the original TADA model for CC data *P*(*x*_ca_,*x*_cn_|*H*_*j*_) using a new model in which case counts were conditioned on total counts: *P*(*x*_ca_|*x*_ca_+*x*_cn_,*H*_*j*_), and we constrained the effect size distribution dispersion parameter (*β*) to prevent an implied proportion of protective variants (Additional file 1: Figures S2 and S3 and Additional file 1: Methods).

extTADA uses a MCMC approach for Bayesian analysis. We extracted posterior density samples from at least two MCMC chains for simulated data and at least three chains for real data. Posterior modes were reported as parameter estimates for all analyses, with 95*%* CIs.

Then, gene-level Bayes factors (BF_gene_) can be calculated for each variant category to compare hypotheses *H*_1_ and *H*_0_ (BF=*P*(*x*|*H*_1_)/*P*(*x*|*H*_0_)). Data could be from heterogeneous population samples; therefore, we extended TADA’s BF_gene_ as the product of BFs of all variant categories including population samples as in

3 $$  \text{BF}_{\text{gene}} = \left[ \prod \limits_{h=1}^{N\text{dn}_{\text{pop}}} \prod \limits_{k=1}^{C\text{dn}} \text{BF}_{dn_{hk}} \right] \left[\prod \limits_{a=1}^{N\text{cc}_{\text{pop}}} \prod \limits_{b=1}^{C\text{cc}} \text{BF}_{cc_{\text{ab}}} \right].  $$

We changed the order of integrals in the BF calculations to avoid numerical integration over *P*(*q*) because the true range of this parameter is not known (Additional file 1). We inferred significant genes by converting BFs to FDRs using the approach of [55] as described in [31]. The posterior probability (PP) for each gene was calculated as PP=*π*×BF/(1−*π*+*π*×BF) [56].

#### Testing the pipeline on simulated data

To test extTADA, we used the simulation method described in the TADA paper [16]. To check the approximate model of CC data, we simulated one CC variant class and two CC variant classes. To check the integrated model for both CC and DN, we simulated one CC and one DN variant class. The original CC model in TADA [16] was used to simulate CC data and then CC parameters were estimated using the approximate model. To make the data more similar to real data, the frequency of SCZ CC LoF variant counts was used to calculate the prior distribution of *q*∼Gamma(*ρ*,*ν*) as described in Additional file 1: Table S3.

Different sizes of samples were used. For CC data, to see the performance of the approximate model, we used four sample sizes: 1,092 cases plus 1,193 controls, 3,157 cases plus 4,672 controls, 10,000 cases plus 10,000 controls, and 20,000 cases plus 20,000 controls. The first two sample sizes were exactly the same as the two sample sizes from the Sweden data in current study. The last two sample sizes were used to see whether the model would perform better if sample sizes were increased. For DN and CC data, we used exactly the sample sizes of the largest groups in our current data sets: 1,077 families, 3,157 cases, and 4,672 controls.

To assess the performance of model parameter estimation, we calculated Spearman correlation coefficients [57] between estimated and simulated parameter values. For each combination of simulated parameters, we reran the model 100 times and used the medians of estimated values. We also used different priors for the hyperparameters (e.g., $\bar {\bar {\gamma }}$ and $\bar {\beta }$ in Additional file 1: Table S3) in the simulation process and chose the most reliable priors corresponding with ranges of $\bar {\gamma }$. Because $\bar {\beta }$ mainly controlled the dispersion of hyperparameters, $\bar {\bar {\gamma }}$ was set equal to 1, and only $\bar {\beta }$ was tested.

To assess the performance of extTADA risk-gene identification, we compared expected and observed FDRs (oFDRs). We defined oFDR as the proportion of FDR significant genes that were true risk genes (determined for data simulation). We simulated DN and CC data for a range of sample sizes, using parameter values randomly sampled from the posterior density of our primary SCZ analysis.

We also conducted power analyses of larger sample SCZ studies using parameters sampled from the posterior density of our primary SCZ analysis. For power analyses, we assumed sample sizes ranging from 500 to 20,000 trio families and equal numbers of cases and controls ranging from 1,000 to 50,000 of each, and calculated the number of risk genes at FDR ≤0.05.

We also tested when there was no signal for both DN mutations and rare CC variants. We simulated one DN category and one CC category with *π*=0 and ${\bar {\gamma } = 1}$. To see the influence of prior information of $\bar {\gamma }$ ($\bar {\gamma } \sim \text {Gamma}(1, \bar {\beta })$) for these results, we used different values of $\bar {\beta }$.

#### Applying extTADA to real data

**Estimating genetic parameters**

For SCZ, we analyzed DN mutations and CC variants from non-heterogeneous population samples. Three DN mutation categories (MiD, LoF, and silentFCPk mutations) and one CC variant category (MiD and LoF variants, pooled) were used in Eq. 2 to obtain genetic parameters for SCZ. Detailed analyses of SCZ data are described in Additional file 1: Methods. We performed exome-wide association analyses with and without covariates to test for stratification, and used clustering to identify non-heterogeneous samples for extTADA analysis. For ASD, two DN (MiD and LoF) and one CC (MiD and LoF pooled) variant categories were analyzed. For the three other disorders, only DN data (MiD and LoF categories) were analyzed because no rare CC data were available.

**Secondary analyses**

We compared our results with those generated using mutation rates adjusted for the ratio of observed to expected synonymous mutations. We divided the observed counts by expected counts (=2×family numbers×total mutation rates), and then used this ratio to adjust for all variant category mutation rates.

We conducted further analyses of the SCZ data. Each variant category (LoF, MiD, silentFCPk DN mutations, and LoF+MiD CC variants) was analyzed individually to assess its contributions to the primary results. We conducted secondary analyses including CC variants present in ExAC, and with equal mean RR parameters ($\bar {\gamma }_{\text {CC}}$ and *β*_CC_) across CC population samples.

**Running** **TADA** **on the current data sets**

We also ran TADA for all the current data sets. To compare the results of extTADA and TADA, TADA was run directly from variant counts as extTADA. We used the method of moments implemented in TADA to estimate *π* and $\bar {\gamma }_{\text {LoF,DN}}$, and then the burden of other variant categories was calculated by dividing case counts by control counts. Gene-level association tests were then conducted as implemented in TADA. The results are shown in Additional file 1: Results, Table S4, and Figure S4.

#### Gene set enrichment in extTADA results

Based on the extTADA results, we tested the enrichment of gene sets by using gene PPs as follows. For each gene, we obtained PP from extTADA. For each gene set tested, we calculated the mean of PPs (*m*_0_). After that, we randomly chose gene sets matched for mutation rates and recalculated mean PP *n* times (*n*= 10 million in this study) (generating the vector *m*). The empirical *p* value for the gene set was calculated as

$$p = \frac{\text{length}(m\left[m > m0\right]) + 1}{\text{length}(m) + 1}. $$

To correct for multiple tests, the *p* values were FDR adjusted using the method of [58]. To match mutation rates, for each gene, we chose random genes from the 1,000 genes with the closest mutation rates.

To test the results of the mean-PP-based method above, we also compared the method with a permutation-based method. For each condition, we chose the top 500 genes with the smallest FDR values from the extTADA results. For each gene set, we calculated the number of overlapping genes between the 500 genes and the gene set (*m*_0_). After that, we randomly chose gene sets having the same length as the tested gene set, and recorded the intersecting gene number with the top 500 genes. This process was carried out *n* times to produce a vector *m* (*n* = 10,000,000). The matching of genes by mutation rate and the empirical *p* value calculation were as described above.

#### Post hoc analysis of significant genes and gene length

Different FDR thresholds were used to test whether significant genes could be affected by gene length. For each FDR threshold, the mean gene length of significant genes (*m*_0_) was calculated. Next, *N* gene sets (*N* = 10,000 in this study) were randomly generated from genes having DN mutations, and their mean gene lengths (*m*) were calculated. The *p* value was calculated as

$$ \frac{\text{length}(m\left[m > m_{0}\right]) + 1}{\text{length}(m) + 1}. $$

#### pLI/RVIS data in novel significant gene sets

Residual variation intolerance score (RVIS) information (RVIS_Unpublished_ExACv2_March2017.txt) was downloaded from [59] and information on the probabilities of LoF intolerance (pLI) was downloaded from [60] on 20 June 2017. To calculate *p*, *μ*, *σ*, and *z* for a gene set, we used the same approach as [41] with 10,000 permutations.

#### Single-cell enrichment analysis

We obtained gene expressions from 9,970 single cells that were previously clustered into 24 different cell types [54]. We used the scran R package [61, 62] using the 50 *%* of the genes with mean expression higher than the median to compute a normalization factor for each single cell. The normalization factors were computed after clustering cells using the scran quickcluster() function to account for cell type heterogeneity. We then performed 24 differential expression analyses using BPSC [63], testing each cell type against the 23 other cell types using the normalization factors as covariates. For each differential expression analysis, the *t*-statistics were then standard normalized. Finally, for each cell type, we tested if the standard normalized *t*-statistic for genes in the gene sets was significantly higher than that for genes not in the gene set.

#### Network and transcriptome analyses

We used GeNets [64] to test protein interactions from the gene sets. Connectivity *p* values were obtained by permuting 75,182 matched random networks, and communities (subnetworks showing greater connectivity within than between) were defined by hierarchical agglomeration [65]. Spatiotemporal transcriptome data were clustered using a hierarchical method inside heatmap.2 of the package gplots [66]. We used a height of 9 (in the function cutree) to divide the data from the clustering results into eight groups. Default options were used for this clustering process. Fisher’s exact test [67] was used to obtain *p* values between spatiotemporal transcriptome clusters and GeNets-based communities.

## Results

### The extTADA pipeline for rare-variant genetic architecture inference

We present a pipeline for integrative analysis of trio-based DN variants and CC rare variants, to infer rare-variant genetic architecture parameters and to identify disease risk genes. We extended the hierarchical Bayesian modeling framework of He et al. [16] to develop extTADA (Additional file 1: Figure S2 and Table S3) for Bayesian analysis via MCMC.

**Evaluating** **extTADA** **on simulated data**

We analyzed simulated DN and CC data with one variant category each and CC data with two variant categories, to examine inference on a single variant class as well as to assess the conditional probability approximation for CC data (Additional file 1: Figures S5–S8, Additional file 1: Results). We tested sample sizes ranging from that of the available data, 1077 trios and 3157 cases (equal controls), and larger sample sizes of up to 20,000 cases (see Additional file 1: Results).

**Fig. 1:**
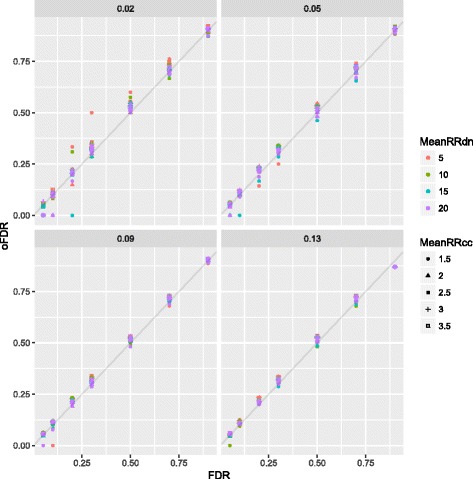
Observed false discovery rates (oFDRs) and theoretical FDR with different combinations between $\bar {\gamma }_{\text {dn}}$ and $\bar {\gamma }_{\text {CC}}$. Each panel is for one *π* value. For example, the top left panel shows oFDR and FDR for *π*=0.02. FDR false discovery rate, dn de novo, FDR false discovery rate, oFDR observed FDR, RR relative risk

We observed little bias in parameter estimation (Additional file 1: Tables S5 and S6). With very large RR of the inherited variants, we observed slight under- and overestimation of the risk-gene proportion ($\hat {\pi }$) and mean RR ($\hat {\bar {\gamma }}$), respectively. We note that these conditions appear outside the range of our SCZ analyses. Some bias can be expected in Bayesian analysis and does not have a large effect on risk-gene identification under this model [16]. We assessed this directly by calculating oFDR, i.e., the proportion of genes meeting a given FDR significance threshold that are true simulated risk genes). extTADA risk-gene identification results were calibrated well (Fig. 1) over wide parameter ranges. For small *π* (e.g., *π*=0.02), oFDRs were higher than FDRs when DN mean RRs ($\bar {\gamma }$) were small (∼5). We also observed oFDRs were equal to zero for some cases with small FDR, when very small numbers of FDR-significant genes were all true risk genes. We also ran extTADA on null data, *π*=0 and $\bar {\gamma } = 1$, for both DN and CC data (Additional file 1: Table S7). Here, MCMC chains tended not to converge, *π* estimates trended to very small values, and BFs and FDRs identified almost no FDR-significant genes as expected (Additional file 1: Table S7).

### Data for analyses

#### Schizophrenia

We applied extTADA to the largest available DN and CC SCZ whole exome sequence data, for inference of rare-variant genetic architecture parameters and for genic association. In total, 6,699 cases, 13,028 controls, 1,077 trio/quad families were analyzed (Additional file 1: Table S1). Primary analyses included three variant categories for DN data (LoF, MiD, and silentFCPk) and a single category of CC singletons [5, 7] not present in the ExAC data (termed NoExAC) [28]: LoF+MiD. An array of secondary extTADA analyses were conducted to help validate and dissect our results.

DN mutations and CC variants were tested to select classes and samples for the extTADA pipeline. For DN mutations, we calculated the sample-adjusted ratios of mutation counts between 1,077 DN cases and 731 DN controls (Additional file 1: Table S1). Like [25], the highest ratio was observed for silentFCPk (2.57), followed by MiD (2.3), LoF (1.83), and missense and silent (∼1.3) mutations (Additional file 1: Figure S9). Three classes (LoF, MiD, and silentFCPk) were used in extTADA analyses.

Since currently extTADA requires integer counts data, adjustment for ancestry and technical covariates is not possible. We performed exome-wide association analyses with and without covariates to test for stratification, and used CC samples to obtain homogeneous population samples (see Additional file 1: Methods). First, for the 4929 cases and 6232 controls from the Sweden population sample, we clustered all cases and controls based on principal components analysis and tested each cluster for CC differences with and without adjustment for covariates. We carried two clusters forward for analysis (groups 1 and 3 in Additional file 1: Figure S10), one with 3,157 cases and 4,672 controls, and the other with 1,091 cases and 1,193 controls. We used only the larger UK population sample from the UK10K project data [8], as it showed comparable CC differences to the homogenous Sweden samples. As in [7], NoExAC singleton CC variants showed significant CC differences and InExAC variants did not (Additional file 1: Figure S10); therefore, we used only NoExAC CC singletons in the primary extTADA analyses. However, we also used all singletons in a secondary analysis for comparison. LoF and MiD variants showed similar enrichment in our CC data (Additional file 1: Figure S10); therefore, we pooled them to maximize the CC information.

#### Neurodevelopmental disorders

The sample sizes for these diseases are shown in Additional file 1: Table S1 and Figure S1. The numbers of trios ranged from 356 for EPI, 1,112 for ID, and 4,293 for DD to 5,122 for ASD. As previously reported (see references in Additional file 1: Table S1), these data have strong signals for DN mutations contributing to disease (Additional file 1: Table S8). Only ASD data included CC samples (404 cases and 3,654 controls) from the Swedish PAGES study of the Autism Sequencing Consortium [31] (see Additional file 1: Methods for details).

### Rare-variant genetic architectures inferred by extTADA

#### Schizophrenia

**Fig. 2:**
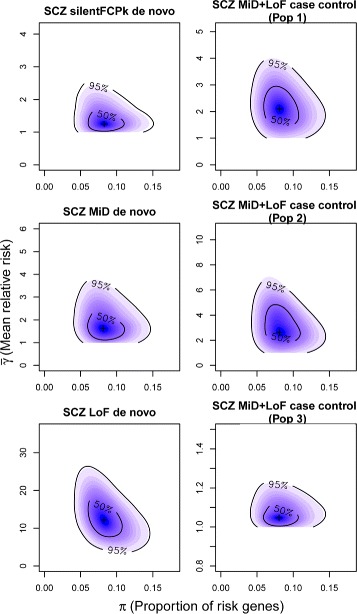
The densities of the proportion of risk genes (*x*-axis) and mean relative risk (*y*-axis) for SCZ data. These were obtained after 20,000 iterations of three MCMC chains. The first two case–control populations are derived from the Sweden data set while the third case–control population is the UK population. The scales on the *y*-axes are adjusted because mean relative risk varies between categories. LoF loss of function, MCMC Markov chain Monte Carlo, MiD missense damaging, Pop population, SCZ schizophrenia, silentFCPk, silent within frontal cortex-derived DNase I hypersensitive site peaks

extTADA generated joint posterior density samples of all genetic parameters for SCZ (Table 1, Fig. 2, and Additional file 1: Figure S11). All MCMC chains showed convergence (Additional file 1: Figure S12). The estimated proportion of risk genes ($\hat {\pi }$) was 8.01*%* of the 19,358 genes analyzed (1,551 genes), with 95% CI (4.59 *%*, 12.9 *%*; 890 to 2,500 genes). DN LoF variants had the highest estimated mean RR ($\hat {\bar {\gamma }}$), 12.25 (95% CI: 4.78-22.22). Estimated mean RRs ($\hat {\bar {\gamma }}$) were 1.22 (95% CI: 1-2.16) for silentFCPk and 1.44 (95% CI: 1-3.16) for MiD. For CC MiD+LoF variants, the two Sweden samples had nearly equal mean RR estimates ($\hat {\bar {\gamma }}$), 2.09 (95% CI: 1.04-3.54) and 2.44 (95% CI: 1.04-5.73), which were larger than that of the UK sample, 1.04 (95% CI: 1-1.19).

**Table 1:** Estimated parameters of proportions of risk genes (pi) and mean relative risk (meanRR) for DN and CC SCZ data and four other NDDs: ID, EPI, ASD and DD

Parameter	Estimated	Lower credible	Upper credible
	mode	interval boundary	interval boundary
SCZ_pi (%)	8.01	4.59	12.9
SCZ_meanRR_silentFCPk_denovo	1.22	1.00	2.16
SCZ_meanRR_MiD_denovo	1.44	1.00	3.16
SCZ_meanRR_LoF_denovo	12.25	4.79	22.22
SCZ_meanRR_MiD+LoF_CCpop1	2.09	1.04	3.54
SCZ_meanRR_MiD+LoF_CCpop2	2.44	1.05	5.73
SCZ_meanRR_MiD+LoF_CCpop3	1.04	1	1.19
ASD_pi (%)	4.44	3.15	5.94
ASD_meanRR_MiDdenovo	3.71	2.06	8.71
ASD_meanRR_LoFdenovo	24.56	14.27	37.44
ASD_meanRR_LoFcc	4.04	2.08	8.24
ID_pi (%)	2.53	1.89	3.43
ID_meanRR_MiDdenovo	29.82	18.86	46.1
ID_meanRR_LoFdenovo	105.45	73.27	143.29
DD_pi (%)	2.84	2.29	3.45
DD_meanRR_MiDdenovo	23.42	13.97	33.97
DD_meanRR_LoFdenovo	88.32	67.54	115.09
EPI_pi (%)	1.14	0.52	2.1
EPI_meanRR_MiDdenovo	72.2	35.39	128.46
EPI_meanRR_LoFdenovo	89.71	45.31	169.43

These results were obtained by sampling three MCMC chains (20,000 times for each chain). These results are for three categories: loss of function (LoF) variants/mutations, missense damaging (MiD) variants/mutations, and silent within frontal cortex-derived DHS peaks (silentFCPk) variants.

*ASD* autism spectrum disorders, *CC* case–control, *DD* developmental disorder, *DN* de novo, *EPI* epilepsy, *ID* intellectual disability, *LoF* loss of function, *MCMC* Markov chain Monte Carlo, *MiD* missense damaging, *NDD* neurodevelopmental disorder, *SCZ* schizophrenia, *silentFCPk* silent within frontal cortex-derived DHS peaks

To test the performance of the pipeline on individual categories and to assess their contributions to the overall results, we ran extTADA separately on each of four single variant classes: silentFCPk, MiD, and LoF DN mutations, and MiD+LoF CC variants (Additional file 1: Table S9). All parameter estimates were consistent with the primary analysis, with broader CIs. The much larger $\bar {\gamma }$ CIs than in integrative analyses demonstrated extTADA’s borrowing of information across data types (also observed in simulation, Additional file 1: Figure S6). To understand convergence in these analyses better, we increased MCMC chain numbers to five for each analysis. LoF DN and MiD+LoF CC chains showed strong convergence, followed by MiD DN. As expected, silentFCPk results (with only 53 mutation counts) showed a lack of strong convergence.

We also assessed the sensitivity of genetic parameter inference in several secondary analyses. We tested extTADA for DN mutations not present in the ExAC database, mutation rates adjusted for the ratio of observed to expected synonymous DN mutations, and an alternative model specification of variant annotation categories. We adjusted mutation rates by a factor of 0.81, the ratio of observed synonymous mutations to that expected based on mutation rates (See ‘Methods’). DN mean RR estimates slightly increased as expected, and the estimated proportion of risk genes increased slightly to 9.37 *%* (95% CI: 5.47-15.12%), while the CC parameters were very similar (Additional file 1: Table S10). Above, we assumed that different CC population samples may have different mean RRs, which could be due to clinical ascertainment, stratification, or population-specific genetic architectures. Analysis using a single mean RR parameter for all three CC samples yielded similar *π* and DNM mean RRs and an intermediate CC MiD+LoF mean RR with a relatively narrower CI, $\bar {\gamma }_{\text {CC}}$ = 1.93 (95 *%* CI 1.08–3.21) (Additional file 1: Table S11 and Figure S13). Considering all CC singleton variants (not just those absent from ExAC) also generated similar genetic parameter estimates, with slightly lower CC mean RRs (Additional file 1: Table S12).

#### ASD, ID, DD, and EPI

**Fig. 3:**
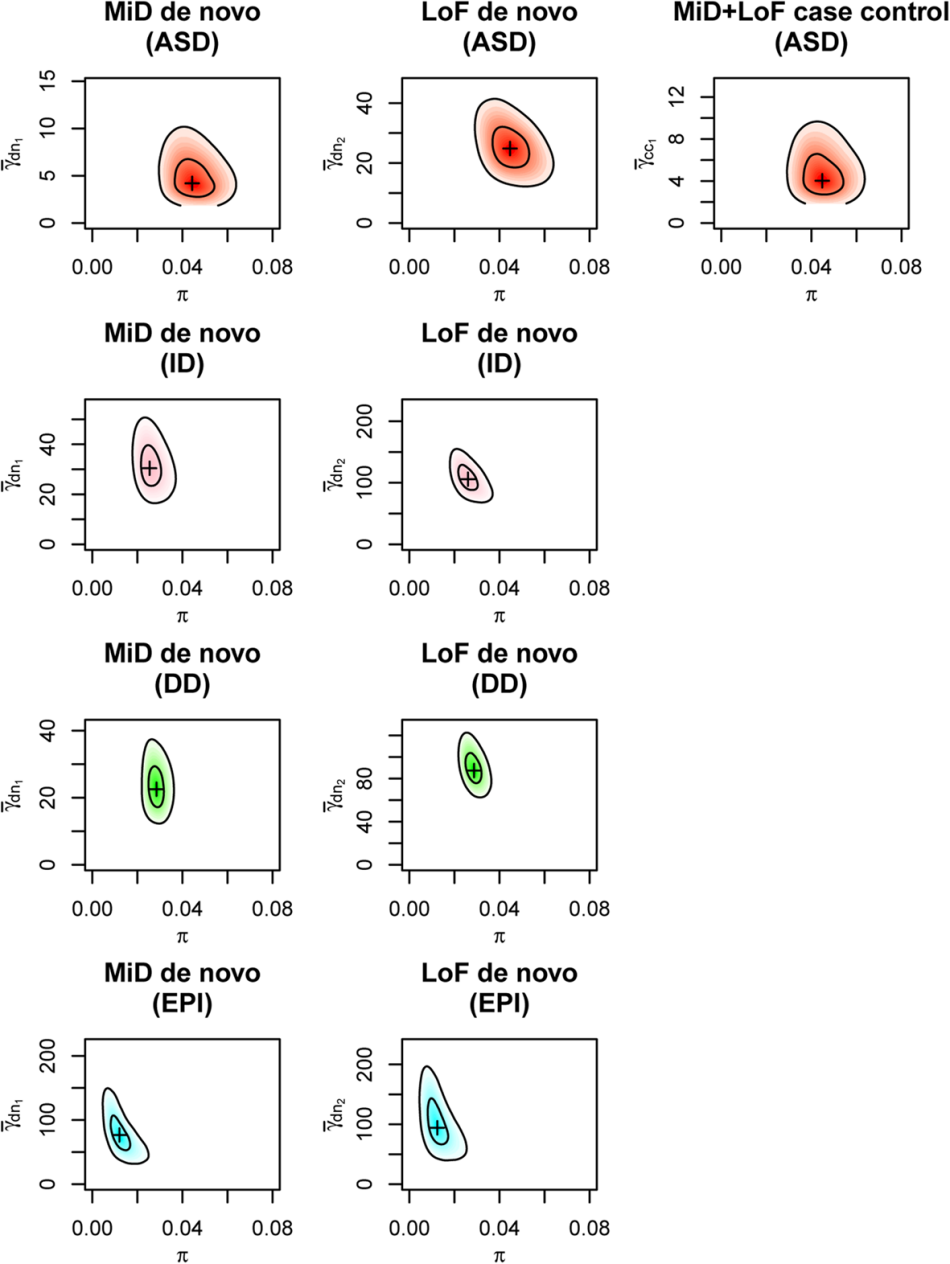
The densities of the proportion of risk genes (*x*-axis) and mean relative risk (*y*-axis) for ASD, EPI, ID, and DD data. These were obtained after 20,000 iterations of three MCMC chains. For ASD, there are two de novo classes and one case–control class. For other disorders, only two de novo classes are publicly available for our current study. The scales on the *y*-axes are adjusted because mean relative risk varies between categories and disorders. ASD autism spectrum disorders, DD developmental disorder, EPI epilepsy, ID intellectual disability, LoF loss of function, MCMC Markov chain Monte Carlo, MiD missense damaging

extTADA genetic parameter estimates are presented in Table 1, Fig. 3, and Additional file 1: Figure S11. MCMC analyses showed good convergence, except for the EPI data with small sample size (356 trios compared with over 1,000 trios for other diseases). Estimated risk-gene proportions ($\hat {\pi }$) for the NDDs were lower than that of SCZ. For ASD, the estimated *π* was 4.44 *%*, (3.15 *%*, 5.94 *%*) or 859 (610–1150) risk genes, consistent with the result of 550–1,000 genes estimated in the original TADA model [16] using only DN LoF data. For DD and ID, the *π* estimates were similar, 2.84 *%* or 550 risk genes (2.29 *%*, 3.45 *%*; 443–668 genes) and 2.53 *%* or 490 risk genes (1.89 *%*, 3.43 *%*; 366–664 genes), respectively, which was smaller than that for ASD. The estimated *π* value for EPI, 1.14*%* or 221 risk genes (0.52 *%*, 2.1 *%*; 101–407 genes), was the lowest but with a broad CI. The estimated mean RRs of DN mutations in all four NDDs were much higher than those of SCZ, indicating a stronger contribution of DN mutations in these four NDDs. For ASD, the estimated mean RRs for DN mutations were consistent with previous results and much lower than for the other diseases. ID and DD had the highest estimated DN LoF mean RRs ($\hat {\bar {\gamma }}$), 105.45 (73.27, 143.29) and 88.32 (67.54, 115.09), respectively. Even though the EPI estimated DN LoF mean RR ($\hat {\bar {\gamma }}$), 89.71 (45.31, 169.43), was similar to those of ID and DD, the estimate for the EPI DN MiD mean RR, 72.2 (35.39, 128.46), was somewhat higher than those of the other diseases. The previously estimated EPI mean RR of 81 [68] is consistent with the current results, and it will be of interest to see if this result remains consistent in additional data in the future.

### Identification of risk genes using extTADA

#### Schizophrenia

Additional file 2: Table S13 includes supporting data as well as association results for SCZ. Four genes achieved PP > 0.8 and FDR < 0.1 (*SETD1A*, *TAF13*, *PRRC2A*, and *RB1CC1*). Two genes, *SETD1A* (FDR = 0.0033) and *TAF13* (FDR = 0.026), were individually significant at FDR < 0.05. *SETD1A* has been confirmed as statistically significant in previous studies [8, 25], while *TAF13* was reported as a potential risk gene only in the study of [6]. However, FDR was high (0.74) for the gene *RBM12*, which was reported as a risk gene for psychosis by [9]. If we increase the FDR threshold to 0.3, as in a recent ASD study, using TADA [31] we identify 24 candidate SCZ risk genes (*SETD1A*, *TAF13*, *RB1CC1*, *PRRC2A*, *VPS13C*, *MKI67*, *RARG*, *ITSN1*, *KIAA1109*, *DARC*, *URB2*, *HSPA8*, *KLHL17*, *ST3GAL6*, *SHANK1*, *EPHA5*, *LPHN2*, *NIPBL*, *KDM5B*, *TNRC18*, *ARFGEF1*, *MIF*, *HIST1H1E*, and *BLNK*). Of these, *EPHA5*, *KDM5B*, and *ARFGEF1* did not have any DN mutations (Additional file 2: Table S13). We note that still more genes show substantial support for the alternative hypothesis over the null model [69] (58 genes with PP > 0.5, corresponding to BF > 11.49, FDR < 0.391; Additional file 2: Table S13). We note that the secondary analyses slightly impacted support for individual genes (Additional file 1: Tables S11 and S12, Additional file 2: Table S14).

#### Neurodevelopmental disorders

The results for the extTADA risk gene of the four disorders ID, DD, ASD, and EPI are presented in Additional file 2: Tables S15–S18. With FDR < 0.05, there were 56, 160, 49, and 9 significant genes for ID, DD, ASD, and EPI. For FDR < 0.1, there were 69, 196, 64, and 10 significant genes.

The genetic parameters inferred after adjusting mutation rates for observed silent DN rates are presented in Additional file 1: Table S10. For ASD, ID, and EPI, the proportions of risk genes were higher than in the primary analyses because the adjustment ratios were less than 1. As a result, the number of significant genes also increased with different FDR thresholds. For DD, the adjustment ratio was >1 (1.16) and the number of significant genes decreased (134 genes with FDR < 0.05). Altogether, 72/134 genes were not among the 93 DD genes reported in a previous study [70], 33 of which were in the list of curated DD genes [71].

We also tested the correlation between gene length and top genes with three different FDR thresholds: 0.05, 0.1, and 0.3. No significant results were observed for these correlations (adjusted *p*≥ 0.25). Only for ASD genes with FDR < 0.05 was a slight gene-size effect observed (unadjusted *p*=0.05, adjusted *p*=0.25, Additional file 1: Table S19).

**Novel significant genes in ID and DD**

The results for the other DN mutation methods using these same data have been recently reported [41, 70]; nevertheless, extTADA identified novel genes with strong statistical support from these recent data.

For ID, we found 56 and 69 genes with FDR ≤ 0.05 and 0.1, respectively. We compared these results with the risk-gene list of [41], which included previously reported and novel ID genes. Altogether, 14 of 56 genes with FDR ≤ 0.05 (*AGO1*, *AGO2*, *ATP8A1*, *CEP85L*, *CLTC*, *FBXO11*, *KDM2B*, *LRRC3C*, *MAST1*, *MFN1*, *POU3F3*, *RPL26*, *TNPO2*, and *USP7*) were not on the list. Of the 14 genes, six (*AGO2*, *CEP85L*, *CLTC*, *FBXO11*, *MFN1*, and *TNPO2*) were strongly significant (FDR < 0.01); these were genes hit by two or three MiD or LoF DNs that were not identified by the analyses of [41]. pLI and RVIS information were obtained for 12 of these 14 genes, and tested using the method of [41]. The median of pLIs was 1 (observed 1; simulated data: *μ*=0.11, *σ*=0.17, *z*=5.08, empirical *p*<9.99×10^−5^). In addition, nine genes (*AGO1*, *AGO2*, *ATP8A1*, *CLTC*, *FBXO11*, *KDM2B*, *MAST1*, *TNPO2*, and *USP7*) had pLI = 1 and one gene (*RPL26*) had pLI = 0.916. The median of the RVISs was −1.49 (observed −1.49; simulated data: *μ*=−0.014, *σ*=0.21, *z*=−7.03, empirical *p*<9.99×10^−5^). Two genes (*CLTC* and *FBX011*) were in the latest list of curated DD genes released on 18 May 2017 [71]. After removing these two genes, pLI was still highly significant (observed median 1; simulated data: *μ*=0.3, standard deviation = 0.39, *z*=1.7, empirical *p* was <9.99×10^−5^), and the RVIS information was not much different (observed −1.48; simulated data: *μ*=−0.01, *σ*=0.23, *z*=−6.26, empirical *p*<9.99×10^−5^).

For DD, there were 160 and 196 genes with FDR ≤ 0.05 and 0.1, respectively. Only 52 of 160 genes with FDR ≤ 0.05 were among the 93 genome-wide significant genes reported by a recent DD study [70] (see below); 98 genes are novel. The 98 genes also included *QRICH1* (FDR = 3.15 ×10^−5^), which was reported as a suggestive DD gene [70]. Like ID, the total MiD+LoF DN counts of these 98 genes were not high (between 2 and 6). Surprisingly, 54 of the 98 novel genes were strongly supported in our results (FDR < 0.01). We assessed the known DD genes in the 93 genes with FDR > 0.05 and saw two common reasons for the differences. Note that we did not analyze the 17 known DD genes on the X chromosome. Most often, our MiD counts were lower than the missense counts of the previous study, since we defined MiD mutations by the intersection of seven prediction algorithms. In addition, extTADA used only the data from 4,293 trios while [70] was a meta-analysis with data from other smaller studies. Still, our results are in agreement with previously published DD gene results (62 of 75 known DD genes on non-chromosome X have extTADA FDR ≤ 0.1; extTADA FDR vs published *P*, Spearman’s *ρ*=0.78, *P*=2×10^−16^).

**Fig. 4:**
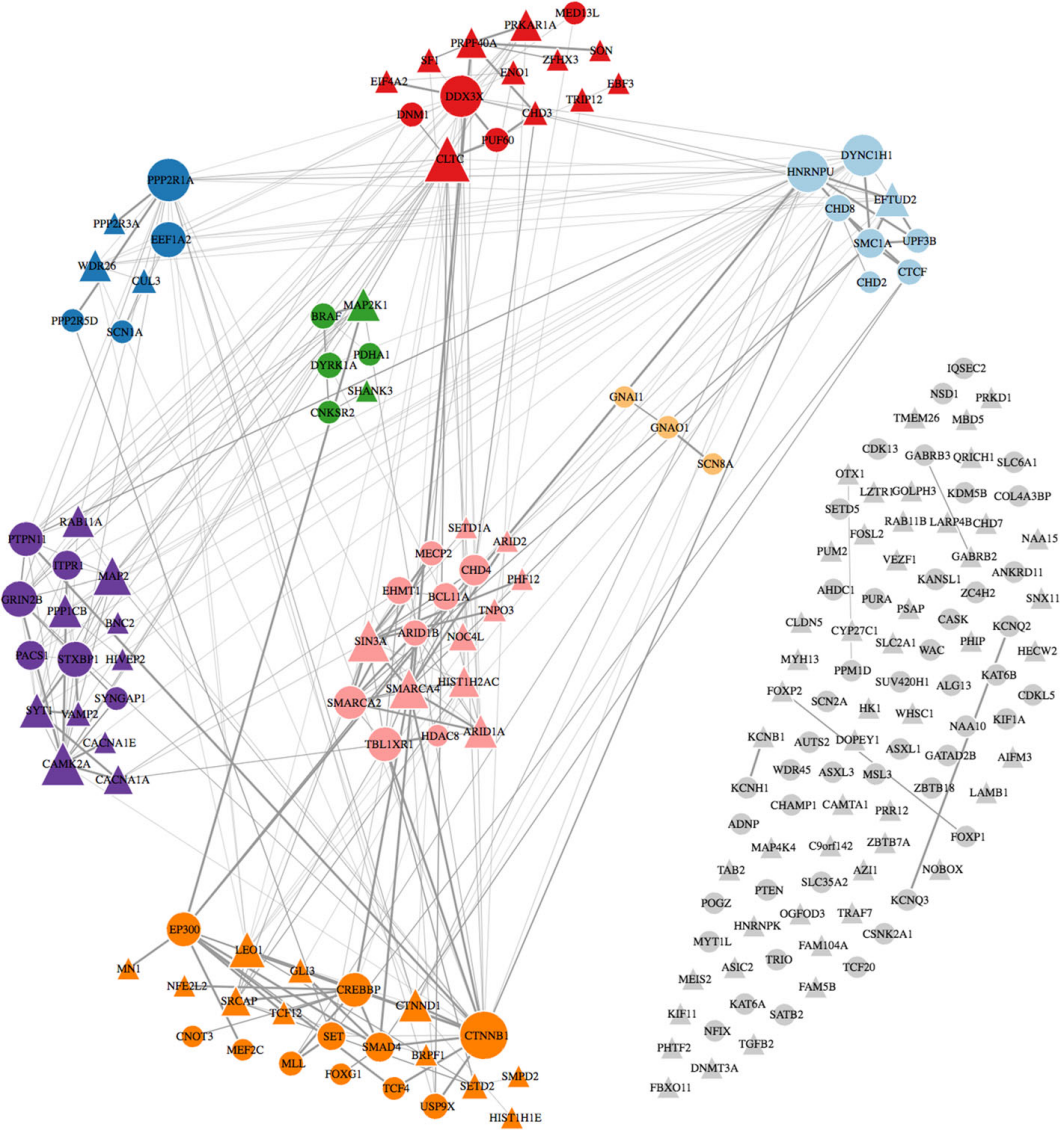
GeNets network analysis for developmental disorder significant genes (*p*<2×10^−3^). These are 93 genome-wide significant genes from [70] and 98 significant genes (FDR < 0.05 from extTADA) not in the 93 genes. Triangular shapes are the 98 novel genes from extTADA. FDR false discovery rate

We sought to validate the large number of novel significant DD genes compared with those of [70] using the same data. First, we compared the enrichment of our candidate gene sets for known DD genes and our novel DD genes. We found that many of the same gene sets were significantly enriched in both previously known and our novel DD genes, with very strong concordance across gene sets (Additional file 1: Figure S14). Altogether, 92 of 98 novel DD genes had pLI and RVIS information. The median pLI was 0.997 (observed 0.997; *μ*=0.033, *σ*=0.036, *z*=26.46, empirical *p*<9.99×10^−5^). The median of the RVISs was −0.92 (observed −0.92, simulated data: *μ*=−0.02, *σ*=0.07, *z*=−11.86, empirical *p* was <9.99×10^−5^). We also found that 43 of the 98 novel DD genes occur in the latest list of curated DD genes (described above), showing that extTADA was able to detect DD genes later identified in other studies. Altogether, 50 of the 55 novel genes not in the curated DD gene list of had pLI/RVIS information. The median of the 50 pLI values was 0.9415 (observed 0.94, simulated data: *μ*=0.045, *σ*=0.064, *z*=13.95, empirical *p* was <9.99×10^−5^). The median of the RVISs was −0.72 (observed −0.72, simulated data: *μ*=−0.01, *σ*=0.10, *z*=−6.87, empirical *p*<9.99×10^−5^). Finally, we used GeNets with the InWeb protein–protein interaction (PPI) network [64] to test the connections between the 98 novel and 93 known genes (191 genes in total). Out of 191 genes, 94 (46 known and 48 novel) were connected to eight communities (overall *p*=0.006, and community connectivity *p*<2×10^−3^) (Fig. 4).

### Power analysis under inferred genetic architecture

**Fig. 5:**
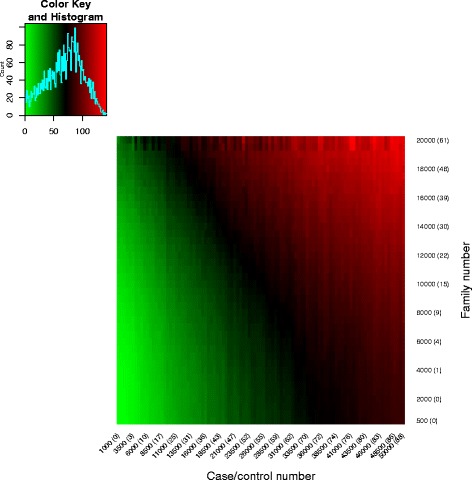
Number of risk genes for different sample sizes based on the genetic architecture predicted by extTADA. Case–control number is only for cases (or controls); therefore, if case–control number = 10,000, this means cases + controls = 20,000. The numbers in brackets show risk-gene numbers if we use only case–control data or only de novo mutation data

We simulated risk-gene discovery using extTADA for the genetic architecture of SCZ inferred from the current data (Fig. 5 and Additional file 1: Figure S15), using the CC population sample with highest mean RR. Samples sizes from 500 to 20,000 trio families and from 1,000 to 50,000 cases (number of controls = number of cases) were simulated as in our validation analyses, using parameters from the posterior distribution samples given the SCZ data. The number of risk genes with FDR ≤0.05 ranged from 0 to 238. Based on this analysis, we expect >50 risk genes for total sample sizes of trio families plus CC pairs of ∼20,000. The results suggest that, assuming sequencing costs are proportional to the number of individuals, generating CC data is more efficient than generating trio data despite the larger relative risk of DN mutations.

### Gene-set enrichment

**Known and novel gene sets are enriched in SCZ risk genes from** **extTADA**

We tested 185 gene sets previously implicated in SCZ genetics or with strong evidence for relevance to SCZ rare variation [5, 7, 15, 39, 42, 68] (Additional file 1: Table S2). FDR-significant results (adjusted *p*<0.05) were observed for 17 gene sets including those previously reported using these data [5–7] (Table 2). The most significant gene sets were missense constrained and LoF intolerant (pLI09) genes, targets of RBFOX1/3 and RBFOX2 splicing factors, CHD8 promoter targets, targets of the fragile X mental retardation protein (FMRP), and CELF4 targets (all *p*<2.0×10^−4^, adjusted *p*≤7.13×10^−3^, Table 2). Genes harboring DN single-nucleotide polymorphisms (SNPs) and indels in DD, and post-synaptic density activity-regulated cytoskeleton-associated (ARC), NMDA-receptor (NMDAR), and mGluR5 complexes were also enriched. Genes exhibiting an allelic bias in neuronal RNA-seq data [39] were also enriched in SCZ extTADA results (*p*=1.9×10^−3^, adjusted *p*=2.58×10^−2^). The two brain RNA-seq co-expression modules derived from the hippocampus [47], M3 and M13, were also significant. Finally, significant enrichment was also obtained for the mouse mutant gene sets with psychiatric-relevant phenotypes including abnormal emotion or affect behavior, abnormal cued conditioning behavior, and abnormal sensory capabilities/reflexes/nociception (FDR < 0.05).

**Table 2:** Enrichment of known gene sets from extTADA results for schizophrenia

Gene set	Gene number	Overlapping gene number	*p* value	FDR
Constrained	1003	939	3.3e-06	6.2e-04
pLI09	3488	3241	1.0e-05	8.2e-04
rbfox2	3068	2895	1.3e-05	8.2e-04
chd8.human_brain	2798	2601	5.0e-05	2.3e-03
rbfox13	3445	3230	1.7e-04	6.3e-03
FMRP_targets	839	792	2.1e-04	6.5e-03
celf4	2675	2468	2.7e-04	7.1e-03
Module.M3	162	145	5.6e-04	1.3e-02
DD.allDenovoMiDandLoF	1271	1271	7.0e-04	1.4e-02
ARC	28	25	1.0e-03	1.8e-02
NMDAR_network	61	58	1.5e-03	2.3e-02
abnormal_emotionORaffect_behavior	392	363	1.5e-03	2.3e-02
AlleleBiasedExpression.Neuron	802	619	1.9e-03	2.6e-02
Module.M13	149	129	2.0e-03	2.6e-02
abnormal_cued_conditioning_behavior	74	67	2.5e-03	2.9e-02
mGluR5	39	36	2.4e-03	2.9e-02
abnormal_sensory_capabilitiesORreflexesORnociception	607	579	4.5e-03	4.9e-02
mir137	3260	2940	7.0e-03	6.5e-02
abnormal_behavior	2037	1937	7.0e-03	6.5e-02
Pardinas2017_extTable9	534	522	7.0e-03	6.5e-02
PSD-95_(core)	65	57	8.0e-03	6.7e-02
abnormal_excitatory_postsynaptic_currents	73	67	8.0e-03	6.7e-02
list.EPI.43genes.2017.Epi4K.2017	43	38	9.2e-03	7.2e-02
abnormal_socialORconspecific_interaction	257	238	9.4e-03	7.2e-02
abnormal_associative_learning	204	190	1.5e-02	1.1e-01
abnormal_social_investigation	64	54	1.8e-02	1.2e-01
Module.M1	1244	1071	1.8e-02	1.2e-01
synaptome	1887	1816	1.9e-02	1.3e-01
abnormal_motor_capabilitiesORcoordinationORmovement	1398	1326	2.0e-02	1.3e-01
CYFIP1_all	37	34	2.1e-02	1.3e-01
abnormal_fearORanxiety-related_behavior	232	213	2.3e-02	1.4e-01
abnormal_behavioral_response_to_xenobiotic	219	208	3.0e-02	1.7e-01
abnormal_learningORmemoryORconditioning	449	414	3.1e-02	1.7e-01
abnormal_brain_size	193	180	3.6e-02	1.8e-01
abnormal_contextual_conditioning_behavior	95	88	3.4e-02	1.8e-01
abnormal_excitatory_postsynaptic_potential	64	58	3.5e-02	1.8e-01
abnormal_aggression-related_behavior	69	62	3.7e-02	1.8e-01
Module.M2	38	35	4.1e-02	2.0e-01
abnormal_discrimination_learning	21	20	4.3e-02	2.0e-01

These *p* values were obtained from 10,000,000 simulations, and then adjusted using the method of [58]. The information for these gene sets is summarized in Additional file 1: Table S2. The second column (Gene number) shows the number of genes in the gene set. The third column shows the number of overlapping genes between the gene sets and the 19,358 genes used by extTADA.

*FDR* false discovery rate

To test more novel gene sets for enrichment in the SCZ extTADA results, we added gene sets from GO, KEGG, REACTOME, C3 from MSigDB [72], and The Mouse Genome Database, filtered for sets including 100–5,000 genes (see ‘Methods’ for details), and FDR-adjusted for the full set of 2,269 gene sets tested (Additional file 1: Table S20). Significant results were observed in eight gene sets including five of the known gene sets. The top known gene sets still had the lowest *p* values in these results. We observed significant enrichment of two C3 conserved non-coding motif gene sets [73]: GGGAGGRR_V$MAZ_Q6, genes containing the conserved M24 GGGAGGRR motif, and ACAGGGT,MIR-10A,MIR-10B, including microRNA MIR10A/B targets; and MP:0005179, decreased circulating cholesterol level less than the normal amount (Additional file 2: Table S20).

#### Multiple gene sets are enriched across NDDs

**Fig. 6:**
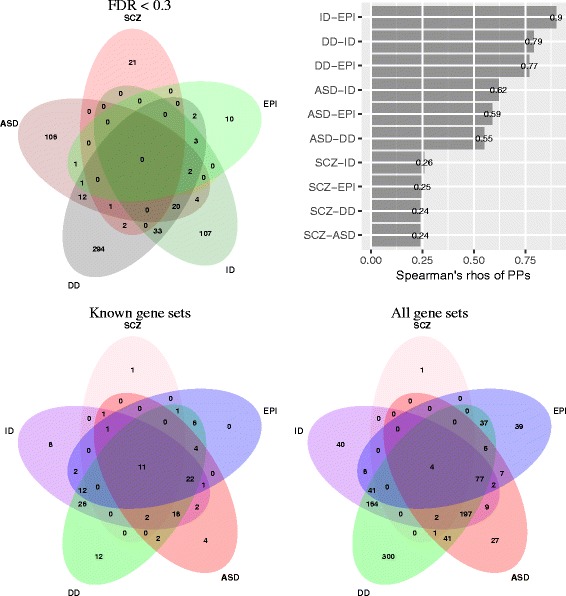
Comparing between five conditions. Top left: Overlaps of top significant genes (FDR < 0.3). Top right: Correlations of posterior probabilities (PPs) between SCZ, ASD, DD, ID, and EPI (all *p* values <0.0001). These results are calculated using PPs from extTADA. Bottom: Overlaps of significant gene sets in SCZ, ASD, EPI, DD, and ID. These results are for 185 and 1,879 gene sets, respectively. ASD autism spectrum disorders, DD developmental disorder, EPI epilepsy, FDR false discovery rate, ID intellectual disability, PP posterior probability, SCZ schizophrenia

We saw above that genes containing DN mutations in several of the diseases studied here are enriched in SCZ extTADA results. We, therefore, tested gene set enrichment in the four NDDs and combined this information with the SCZ gene-set information above (Additional file 2: Tables S21 and S22). Of the 185 known or strong-candidate gene sets tested in SCZ, 106, 116, 68, and 60 gene sets were significant (FDR < 0.05) for ID, DD, ASD, and EPI, respectively. There were 11 gene sets that were significant across all five diseases: constrained, PLI09, rbfox2/13, FMRP targets, CELF4, ARC, NMDAR network, abnormal emotion/affect behavior, abnormal sensory capabilities/reflexes/nociception, abnormal excitatory postsynaptic currents, and hippocampus co-expression module M3 [47]. The significant result of genes in M3 replicated the result of [47]. However, we note that many more gene sets were significant across two or more NDDs, but not SCZ (Fig. 6). Our broader set of 2,269 gene sets showed a similar pattern of sharing; there were only four gene sets that were significant (FDR-adjusted *p*<0.05) in all five diseases, while many more gene sets were significant across two or more NDDs (Fig. 6).

To validate the gene-set results above, we tested gene-set enrichment using the number of genes in the gene set that were in the extTADA top 500 genes. We saw high correlations between the PP-mean-based approach above and this approach (Additional file 1: Figure S16).

### Network facilitated interpretation of NDD risk genes

**Overlap among NDD** **extTADA** **results**

There was no gene significant across SCZ and the four NDDs with FDR < 0.05 or 0.1. Only *SCN2A* was significant across the four NDDs with these thresholds, but was not in SCZ (FDR = 0.35). This gene has been reported as a strong risk gene for multiple NDDs (reviewed in [2]). Only one additional gene, *STXBP1*, was significant across the four NDDs when the threshold FDR was increased to 0.3 and it was not significant for SCZ (FDR = 0.9). At FDR < 0.3, several genes were shared among two or three NDDs, whereas only three genes were shared between SCZ and any NDD (Fig. 6). We also calculated the correlations between risk-gene PPs for all diseases. Interestingly, high correlations were observed for the four NDDs (*ρ*>0.5) but not for SCZ and the NDDs (*ρ*<0.3, Fig. 6), either for all genes or for significant/suggestive genes in any disease. The pattern of sharing of top extTADA results across diseases was consistent when examining gene set enrichment (Fig. 6).

Given the high level of sharing among neurodevelopmental disease risk genes and the large number of novel significant genes we identified, we undertook network analyses to assess and interpret the neurodevelopmental disease risk genes. We chose 288 NDD genes with different FDR thresholds to balance the number of significant genes across the four NDDs. These thresholds were 0.05 for DD, 0.1 for ASD and ID, and 0.5 for EPI.

**Fig. 7:**
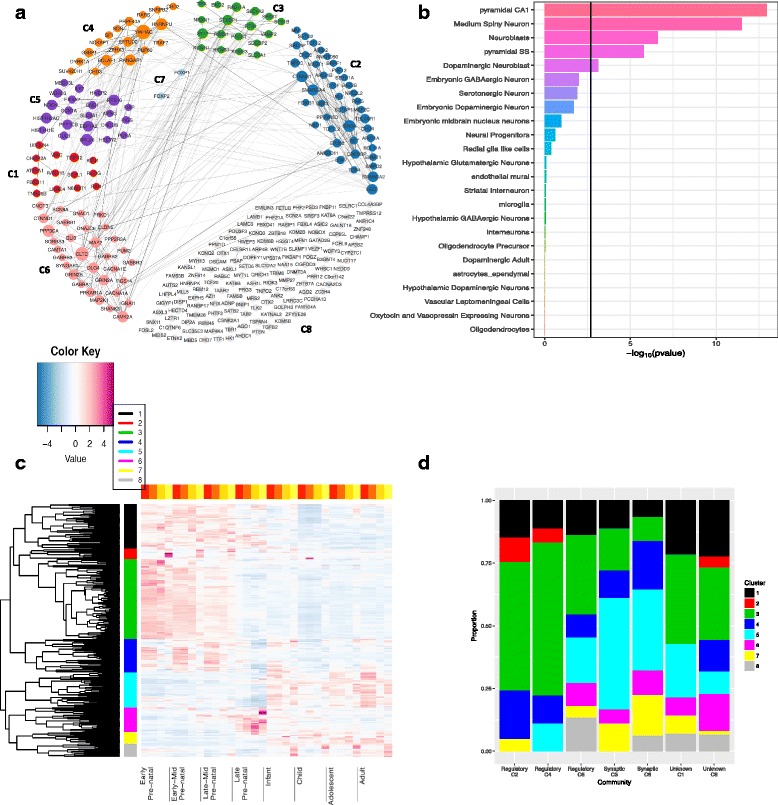
Analyzing results for 288 neurodevelopmental disorder genes. **a** GeNets results for the top 288 neurodevelopmental disorder genes. Here, 149/288 genes were connected into seven main communities (colored genes) and the unconnected genes were put into the eighth community. **b** Enrichment of the 288 genes in different cell types. **c** Grouping the 288 genes to distinct spatiotemporal expression. Genes were clustered into eight groups using a hierarchical clustering method (color bar). **d** The proportions of different clusters in the eight communities

First, we used GeNets [64] to test for significant connectedness and structure of NDD genes in the InWeb PPI network. Including second-degree indirect connections, the 288 NDD genes were connected with 89 candidate genes to make a network of 377 genes. These 377 genes were connected in seven communities (subnetworks, C1–C7), including 149 of the 288 NDD genes (overall connectivity *p* value and connectivity *p* values for each community <1.3×10^−5^, Fig. 7 and Additional file 2: Table S23). Canonical pathway enrichment was observed for five communities, suggesting that they are functionally distinct. Significant pathways included beta-catenin nuclear signaling, transcriptional regulation of white adipocyte differentiation, WNT signaling pathway, and circadian clock (C2); release of several neurotransmitters (C3); spliceosome (C4); ribosome and 3 ^′^ UTR-mediated translational regulation (C5); and neurotransmitter receptor binding and downstream transmission in the postsynaptic cell, calcium signaling, and post NMDA receptor activation events (C6) (Additional file 2: Table S24). Similar results were obtained on restricting the network to direct edges only (connectivity *p*<0.002, Additional file 1: Figure S17), although the resulting 12 communities were less functionally distinct in pathway enrichment.

Second, we used mouse single-cell RNA-seq data [54] to test NDD gene enrichment across brain cell types. Significant results were observed for hippocampal CA1 pyramidal cells (*p*=1.6×10^−9^), followed by neuroblasts, medium spiny neuron cells, somatosensory pyramidal cells, and dopaminergic neuroblasts (*p*<6.6×10^−4^, Fig. 7). We further tested each GeNets PPI community separately (Additional file 1: Figure S18), and found multiple cell types enriched in five communities, C2–C6, consistent with their regulatory or synaptic pathway enrichment. Specifically, C2, C4, and C5 were significantly enriched in neuroblasts and neural progenitor cells while C3 and C6 were enriched for pyramidal CA1 and SS cells (among a few others).

Third, we used BRAINSPAN RNA-seq data to cluster the 288 genes based on their spatiotemporal expression in the developing brain (Fig. 7). The genes clustered into eight groups, and again correlated with PPI communities. Genes in prenatally expressed groups (clusters 1, 3, and 4) were overrepresented in regulatory communities C2 and C4 (*p*=3.78×10^−5^). Postnatally expressed groups (clusters 5, 7, and 8) were in higher proportions in the synaptic communities C3 and C6 (*p*=1.42×10^−7^).

## Discussion

In this work, we built a pipeline, extTADA, for the integrated Bayesian analysis of DN mutations and rare CC variants to infer rare-variant genetic architecture parameters and identify risk genes. We applied extTADA to data available for SCZ and four other NDDs (Additional file 1: Figure S1).

**The** extTADA **pipeline**

extTADA is based on previous work in autism sequencing studies, TADA [16, 31]. It conducts a full Bayesian analysis of a simple rare-variant genetic architecture model and it borrows information across all annotation categories and DN and CC samples in genetic parameter inference, which is critical for sparse rare-variant sequence data. Using MCMC, extTADA samples from the joint posterior density of risk-gene proportion and mean relative risk parameters, and provides gene-level disease-association BFs, PPs, and FDRs. We hope that extTADA (https://github.com/hoangtn/extTADA) will be generally useful for rare-variant analyses across complex traits. extTADA can be used for rare CC variant and/or DN mutation data. The current TADA version uses multiple steps or requires prior information for genetic parameters [8, 74, 75], while extTADA jointly estimates all parameters in a single step without requiring any prior information. If multiple variant categories are used and at least one has a high mean RR, then the parameter results can be accurate for a range of sample sizes (Additional file 1: Figures S6 and S7).

The inference of rare-variant genetic architecture is of great interest in its own right [76], but of course risk-gene discovery is a primary objective of statistical genetics. We have shown how the two are not separable through a power analysis of larger sample numbers under the inferred genetic architecture parameters (Fig. 5). These analyses, incorporated into extTADA, show how study design should be influenced by an analysis of currently available data.

As in all Bayesian and likelihood analyses, we must specify a statistical model; the true model underlying the data is unknown and could in principle yield different results. This is addressed by analyzing a simple model that can allow illustrative, interpretable results, and by assessing sensitivity to alternative model specifications. extTADA uses relatively agnostic hyper-parameter prior distributions (Additional file 1: Figure S2), without previously known risk-gene seeds. extTADA assumes that different variant classes share risk genes such that the mixture model parameter *π* applies to all data types, facilitating borrowing of information across classes. This is supported by convergent DN and CC rare-variant results in SCZ [5–8] (Additional file 1: Table S9); however, some evidence exists for disjoint risk genes for DN vs CC protein-truncating variants e.g., in congenital heart disease [77]. We assume Poisson-distributed counts data and Gamma-distributed mean RR across genes for analytical convenience. The Poisson distribution is likely to approximate genetic counts data well [16], assuming linkage disequilibrium can be ignored and that stratification has been adequately addressed. Poisson DN counts further assume known mutation rates; in our data, mutation rate adjustment for silent DN rates was actually anti-conservative (except for DD). Differences between DN studies are not unlikely even though previous studies of [8, 31] did not adjust mutation rates to account for it. Additional limitations include that we are using public data sets from different sequencing centers, with different technologies and coverages. Thus, although we developed extTADA to utilize summary counts data, care must be taken to avoid sample heterogeneity, particularly when individual-level data are not available. The ability to incorporate covariates, perhaps by modeling Gaussian sample frequency data, would be an important further extension of TADA-like models. In this study, BFs and FDRs are used to obtain the statistical significance of a gene. These measurements can be converted to *p* values using a simulation-based method implemented in the TADA package. A detailed explanation of this approach was presented in [16].

**Insights for SCZ**

The current study generally replicated previous studies and generated new insights for SCZ. In this study, we described in detail the rare-variant genetic architecture of SCZ. It appears more complex than those of ASD, ID, DD, and EPI; the estimated number of SCZ risk genes, ∼1,551, is higher than those of the four other NDDs, and their RR is weaker (Figs. 2 and 3, Table 1). Based on our inference, we showed that tens of thousands of samples are required to identify many rare-variant risk genes (≥50) [76], and that, in contrast to autism studies [16, 31], CC studies may be more efficient than trio studies in risk-gene identification. We found that *SETD1A* [8, 25] is the most significant gene across analyses (FDR ∼1.5×10^−3^), and that *TAF13* [6] is FDR significant. Of two genes with 0.05< FDR <0.1, rare duplications covering *RB1CC1* have been reported in SCZ [78] and in ID and/or DD [79]. Two novel conserved non-coding motif gene sets showing brain-specific expression [73] were enriched (Additional file 1: Table S20), including targets of the transcription factor MAZ and of microRNAs MIR10A/B. In addition, we see a slight overlap between rare and common variant genes [15] (*p*=0.007, FDR = 0.06).

**Insights for NDDs**

We used extTADA to infer genetic parameters for four other NDDs: ASD, EPI, DD, and ID (Table 1, Fig. 3). The ASD results from extTADA are comparable to previous results [16, 31]. We found lower risk-gene proportions particularly for DD and ID, and exceptionally high DN MiD mean RR estimated for EPI (also consistent with previous analyses [80]). The small estimated *π* and large RR ($\hat {\bar {\gamma }}$) facilitated the identification of novel risk genes, particularly for DD. We did not restrict our primary analyses to private DN mutations (not in ExAC) as recently discussed [81]; however, we note that mutation rate calibration might be required for analyses focusing on private mutations. Nonetheless, multiple ID/DD genes discovered in this study are in lists of curated ID/DD genes. In addition, our novel significant genes have similarly high conservation (e.g., pLI and RVIS), like recently discovered ID/DD genes [41]. This shows that using both private and non-private DN mutations provide power for finding significant genes. One might expect that the large estimated proportions of risk genes (*π*) might correspond to large mutational targets for disease risk and substantial common SNP heritability estimates, as observed for ASD and SCZ [82, 83]; however, the large reported SNP-heritability for EPI [84] seems an exception to this pattern, and data for more disorders may better inform this hypothesis. We also highlight the sharing of risk genes across the NDDs (Fig. 6). Multi-phenotype analyses leveraging this sharing could have higher power for detecting novel risk genes.

We conducted network analyses of 288 top NDD risk genes from extTADA. We identified highly significant PPI connectivity and communities differentially enriched for functionally distinct canonical pathways (Fig. 7 and Additional file 2: Table S24). A substantial number of the genes found are synaptic, and particularly present in communities C3 (presynaptic) and C6 (postsynaptic).

The presynaptic PPI community identified in this study (C3, Fig. 7) accumulates genes for which synaptic phenotypes are particularly strong in null mutant mice (*STXBP1*, *STX1B*, *SYT1*, *RIMS1*, and *VAMP2*). *STXBP1*, the only significant gene across the four NDDs (FDR < 0.3), is involved in preparing synaptic vesicles for regulated secretion (reviewed in [85]). The stxbp1 (munc18-1) null mutant shows a loss of all aspects of synaptic transmission [86] and it is the strongest phenotype among all mutants described to date for presynaptic genes. The loss of one copy of the gene in mice leads to subtle synaptic defects [87], which are more severe in inhibitory neurons than in excitatory neurons [87]. Therefore, this implicates an excitation/inhibition imbalance, a central aspect in EPI pathogenesis, which is implicated also in autism and SCZ [88]. Known clinical features of DN heterozygous *STXBP* mutations (reviewed in [89]) include severe ID, seizures, and autistic traits [89].

Of the postsynaptic density proteins, C6 includes the prerequisite glutamate-gated ion channel-forming subunit GRIN1 of the NMDA receptor complex. In contrast to AMPA-type glutamate receptor subunits, which are not present, NMDARs are important for Ca-dependent signaling and plasticity processes. The Ca-dependent calmodulin kinase II (CAMK2A) and phosphatase PPP3CA are also identified as NDD risk genes in C6. Interestingly, *PPP3CA* has just been recently identified as a novel epileptic encephalopathy gene [90]. Other important protein phosphatases are found in different communities: PPP1CB in C5 and PPP2R5D in C2. Mutations in these Ca-mediated signaling proteins are well known to affect synaptic plasticity and lead to major neuronal dysfunction [91–95].

The postsynaptic community C6 also contains the three GABA-binding beta subunits (GABRB1-3) of the GABAA receptor (out of the myriad of GABAA receptor subunit diversity), G-protein coupled receptor signaling (GABBR2, RGS14, and GNAO1), cell adherence-mediated signaling (CNNTD1 and CNNTB1 in C2), and the major postsynaptic density protein-interaction scaffold organizing proteins DLG4, SHANK3, and SYNGAP1, mutants of which have been shown to have a major impact on synaptic function [96, 97]. Also notable among the 288 NDD risk genes are ion channels with roles in excitability including calcium channel subunits CACNA1A/1E (C6); the auxiliary calcium channel subunit CACNA2D3 (C8); three pore-forming sodium channel subunits, SCN8A (C6), SCN1A (C5), and the well-known strong NDD risk gene SCN2A (C8); and potassium channel subunits KCNQ2/3 (C8) [98]. Finally, transcriptional activator AUTS2 occurs in unconnected C8 and is a candidate for NDDs including ASD, ID, and DD [99].

In single-cell RNA-seq data, the top enriched cell types were CA1 pyramidal cells and striatal medium spiny cells, similar to SCZ [54]. In contrast to SCZ, neuroblasts and neural progenitor cells were also clearly enriched for NDDs. Enrichment in neuroblasts and neural progenitor cells was driven by PPI communities (C2, C4, and C5) enriched in regulatory pathways, while enrichment in neurons was driven by the synaptic communities (C3 and C6) (Additional file 1: Figure S18). Expression of NDD genes across development correlated with PPI communities and scRNA-seq enrichment. The majority of the 288 NDD genes are expressed in the brain prenatally [100–102], particularly genes in regulatory PPI communities [103, 104]. Multiple NDD genes are also expressed across development stages [105], including those in synaptic communities. These analyses reveal that different cellular machinery is involved in NDD etiology, and together with the occurrence of at least some known interactors across PPI communities (see above), this suggests that even synaptic proteins confer risk in pre- and postnatal stages of development, perhaps through as yet unknown mechanisms.

**Limitations of the current study**

There are limitations of the current study. First, there are inherent limitations to model-based analyses, as noted above. Second, we used limited variant annotation categories based on our previous studies [7, 16, 25]; we did not use all non-synonymous DN mutations [6, 70], contributing to the differences between our significant DD genes and previously published results [70], and did not ExAC-filter DN mutations [81]. As with any genetic analysis, our findings should be replicated and validated in future studies. Finally, the current sample sizes are not large: only approximately 1,000 trios for SCZ and ID, and only 356 for EPI, resulting in broad CIs. The EPI parameters in particular did not show strong convergence (which may increase sensitivity to prior distributions). Future studies with more comprehensive sets of variant categories and larger sample sizes are likely to improve the current findings.

## Conclusions

We have developed the extTADA pipeline and analyzed rare variants in SCZ and four NDDs. For SCZ, we generated new insights particularly for rare-variant genetic architecture. It is more complex than the four other NDDs with a larger risk-gene proportion. For developmental delay (DD), 98 new significant genes were identified and validated in silico. These genes are highly connected with previous DD genes in a PPI network, and have similar conservation and gene set enrichment to known DD genes. To understand NDD genes better, we further analyzed 288 top NDD genes from extTADA. PPI network analysis shows that these genes are strongly connected in functionally distinct subnetworks based on canonical pathway enrichment, single-cell RNA-seq cell types, and developmental transcriptomic data, revealing some of the most important players and processes dysregulated in NDDs.

## Additional files

Additional file 1 Supplementary Information. This file describes supplementary results, methods, data, figures, and short tables. (PDF 3610 kb)

Additional file 2 Supplementary Tables. This file consists of long supplementary tables. (XLSX 13200 KB)

## Abbreviations

ASD: Autism spectrum disorders
BF: Bayes factor
CC: Case–control
CI: credible interval
DD: Developmental disorder
DHS: DNase I hypersensitive site
DN: de novo
ExAC: Exome Aggregation Consortium
extTADA: Extended Transmission and De novo Association
EPI: Epilepsy
FDR: False discovery rate
HAR: Human accelerated region
ID, Intellectual disability; InExAC: Inside ExAC
LoF: Loss of function
MCMC: Markov chain Monte Carlo
MiD: Missense damaging
NDD: Neurodevelopmental disorder
NIH: National Institutes of Health
NoExAC: Not inside ExAC
PAR: Primate accelerated region
oFDR: observed false discovery rate
pLI: Loss-of-function intolerant
PP: Posterior probability
PPI: Protein– protein interaction
RR: Relative risk
RVIS: Residual variation intolerance score
SCZ: Schizophrenia
silentFCPk: Silent within frontal cortex-derived DHS peaks
SNP: Single-nucleotide polymorphism
