# Supplementary material for: Integrated Bayesian analysis of rare exonic variants to identify risk genes for schizophrenia and neurodevelopmental disorders
Source: Genome Med. 2017 Dec 20;9:114. doi: 10.1186/s13073-017-0497-y (PMC5738153; doi:10.1186/s13073-017-0497-y)
Supplement: Supplementary file 1 — Supplementary Information. This file describes supplementary results, methods, data, figures, and short tables. (PDF 3610 kb) [file 13073_2017_497_MOESM1_ESM.pdf]

Integrated Bayesian analysis of rare exonic variants to identify risk genes for schizophrenia and neurodevelopmental disorders

December 12, 2017

Contents

1 Additional file 1 — Supplementary Information 1

1.1 Supplementary Results 1

1.1.1 Comparing genetic parameters between `extTADA` and `TADA` 2

1.2 Supplementary methods 3

1.2.1 Analysis of SCZ data 3

1.2.2 `extTADA` pipeline: extended transmission (case-control) and de novo analysis 4

1.2.3 Infer parameters using MCMC results 6

1.3 Supplementary Figures 8

1.4 Supplementary Tables 26

1 Additional file 1 — Supplementary Information

This file (AdditionalFile1.pdf) describes supplementary results, methods, data, figures and short Tables.

1.1 Supplementary Results

1.1.0.1 Simulation case-control data only

To evaluate the performance of the approximate CC model for different parameter values, we simulated a single CC sample with either one or two variant/annotation classes. We tested sample sizes ranging from that of the available data,

1,092 each cases and controls (ASD), and 3,157 cases and controls (SCZ), to larger sample sizes of 10,000 cases and controls, and 20,000 cases and controls.

Overall, high correlations (~ 1) between estimated and simulated parameter values indicate little bias in inference based on CC data (Figure S5 and S7). Slight over estimation was observed for the sample size of 1092, especially for risk-gene proportions.

An additional analysis was carried out to assess the performance of specific simulated values. Correlations were calculated for each mean RR and π value. For one CC class, mean RRs were estimated well by the model with correlations ~ 1 (Figure S6). However, the proportion of risk genes was affected by mean RRs. They were estimated well when mean RRs were between 1.5 and 3.5, but underestimated with smaller mean RRs and slightly overestimated with larger mean RRs (Figure S6). For two CC classes, high correlations (≥ 0.97) between simulated and estimated values were seen for all parameters. In addition, small mean RRs of a given class did not directly affect the estimated values of proportions of risk genes (Figure S8).

The issue of poor estimation for one class, but good estimation for $>$ one class was expected. This was an advantage of using multiple classes compared to using only one class in the estimation process when the clustering signal was not very strong. Small mean RRs could result in difficulties in the calculation process to differentiate between a risk gene (mean RR > 1) and a non-risk gene (mean RR ~ 1). If one class was used then many risk genes would be considered to be non-risk genes. If more than one class was used, such risk genes would be assigned as genuine risk genes due to the information available from other classes.

1.1.1 Comparing genetic parameters between `extTADA` and `TADA`

We also applied the current version of `TADA` to SCZ and four NDDs. Estimated risk-gene proportions ($\hat{\pi}$) were smaller than those of `extTADA`, except for DD (Table 1.4). For SCZ, EPI, DD, these estimates were within the CIs of `extTADA`. However, estimated π values were too small for ASD and ID, only 1.8% and 1% respectively. ASD's $\hat{\pi}$ was also much smaller than the result reported by `TADA` using a smaller data set (He et al., 2013). `TADA` mean RR estimation ($\hat{\bar{\gamma}}$) did not converge for MiD, silentCFPk *de novo* mutations as well as the third CC population sample for SCZ. This might be because the genetic signal of these categories was not very strong. For four NDDs, estimated mean RRs ($\hat{\bar{\gamma}}$) were higher than those of `extTADA`, although mostly within the `extTADA` credible intervals. We would expect these differences between two pipelines because we only used variant-count information to input for `TADA`, and only LoF de novo mutations to estimate π; if more prior information were used, the `TADA` results might converge to inside CIs of `extTADA`. Importantly, we note that the parameter estimation procedure for `TADA` does not provide confidence intervals, which is a main motivating rationale for the development of `extTADA`. Finally, the differences in estimated parameters yield different, although overall strongly correlated, gene-level association results (Figure S4).

1.2 Supplementary methods

1.2.1 Analysis of SCZ data

1.2.1.1 Obtaining non-heterogeneous population samples for case-control data of SCZ

The case-control data sets were divided into three big populations: Finland, United Kingdom and Sweden. For the Sweden population, this was a large data set and was also sequenced at different centers (Genovese et al., 2016), therefore we divided this population as follows.

A simple combination between a clustering process using a multivariate normal mixture model and a data analyzing strategy using linear and generalized linear models was used to divide the Sweden data into non-heterogeneous populations. Genovese et al. (2016) recently analyzed all case-control data sets by adjusting for multiple covariates: genotype gender of individuals (SEX), 20 principal components (PCs), year of birth of individuals (BIRTH), Aligent kit used in wet-labs (KIT) by using linear regression and generalized linear regression models as in Equation 1. They reported significant results for NoExAC LoF and MiD variants; therefore, this information was used in this step. We defined homogeneous populations as populations which were not much affected by the covariates. Thus, for the populations, analyzing results using Equation 1 (adjusting covariates) would not be much different from those results using Equation 2 (not adjusting covariates). The `mclust` package Version 5.2 (Fraley and Raftery, 1999) which uses a multivariate normal mixture model was used to divide 11,161 samples (4,929 cases and 6,232 controls) into different groups. To see all situations of the grouping process, we used `mclust` with three strategies on 11,161 samples: grouping all 20 PCs, grouping all 20 PCs and total counts, and grouping only the first three PCs. The number of groups were set between 2 and 6. For each clustering time, Equation 1 and 2 were used to calculate p values for each variant category of each group from the clustering results (p1 and p2 respectively); then, Spearman correlation (Spearman, 1904) between p-value results from the two Equations (cPvalue) was calculated. Next, to filter reliable results from the clustering process, we set criteria:

- cPvalue ≥ 0.85 and p-values for NoExAC ≤ 0.005.
- Ratio p1/p2 from Equation 1 and 2 had to between 0.1 and 1.

From results satisfied the above criteria, we manually chose groups which had similar results between Equation 2 and 1.

$$
\begin{aligned}
&logit(P(SCZ = 1)) \sim count + countAll + sex + birth + kit + \sum_{i=1}^{20} PC_i \\
&count \sim SCZ + countAll + sex + birth + kit + \sum_{i=1}^{20} PC_i
\end{aligned}
\tag{1}
$$

$$\begin{aligned} SCZ &\sim count \\ count &\sim SCZ \end{aligned} \quad (2)$$

For the data from the UK10K project (Singh et al., 2016), we divided the data into two separate populations England and Finland, and tested NoExAC variants in these populations by calculating sample-size-adjusted ratios between cases and controls. The ratios were 0.91 and 0.95 for the UK data. Regarding the Finland data, the ratio for MiD variants was only 0.41 which were extremely low. This could be a special case for the population or might be because of other technical reasons. We did not use this population in the next stage because it showed a different trend with other populations.

1.2.2 `extTADA` pipeline: extended transmission (case-control) and de novo analysis

This section describes more details the pipeline.

1.2.2.1 `extTADA` for one *de novo* population and one case/control population

`extTADA` is summarized in Table S3 and Figure S2. There,
$x_{dn} \sim Pois(2N_d\mu, \gamma_{dn}), x_{ca} \sim Pois(qN_1\gamma_{cc})$, $x_{cn} \sim Pois(qN_0)$, and
$\gamma_{dn} \sim Gamma(\bar{\gamma}_{dn}\beta_{dn}, \beta_{dn})$, $\gamma_{cc} \sim Gamma(\bar{\gamma}_{cc}\beta_{cc}, \beta_{cc})$, $q \sim Gamma(\rho, \nu)$.

Let K be the number of categories (e.g., LoF, MiD), and $x_i = (x_{i1}, .., x_{iK})$ be the vector of counts at the ith given gene. The Bayes Factor for each jth category to test two hypotheses: $H_0 : \gamma = 1$ versus $H_1 : \gamma \neq 1$ was:

$$\begin{aligned} B_{ij} &= \frac{P(x_{ij}|H_1)}{P(x_{ij}|H_0)} \\ &= \frac{\int P(x_{ij}|\gamma,q)P(q|H_1)P(\gamma|H_1)dqd\gamma}{\int P(x_{ij}|\gamma,q)P(q|H_0)P(\gamma|H_0)dqd\gamma} \\ & Because\ \gamma = 1 for\ H_0 \\ &= \frac{\int P(x_{ij}|\gamma,q)P(q|H_1)P(\gamma|H_1)dqd\gamma}{\int P(x_{ij}|q)P(q|H_0)dq} \end{aligned} \quad (3)$$

In Equation 3, $x_{ij} = x_{dn}$ for *de novo* data and $x_{ij} = (x_{ca}, x_{cn})$ for case-control data. In addition, the integral over q was not applicable for *de novo* data because there is no q parameter for *de novo* data.

As in He et al. (2013), the BF for the ith gene combining all categories is:

$$B_i = \prod_{j=1}^{K} B_{ij} \quad (4)$$

To calculate BFs, hyper parameters in Table S3 need to be inferred. Let ϕ_{1j} and ϕ_{0j} be hyper-parameters for H_1 and H_0 respectively. A mixture model of

the two hypotheses was used to infer parameters using information across the number of tested genes (m) as:

$$P(x|\phi_1, \phi_0) \quad = \prod_{i=1}^{m} \left[\pi \prod_{j=1}^{K} P(x_{ij}|\phi_{1j}) + (1-\pi) \prod_{j=1}^{K} P(x_{ij}|\phi_{0j}) \right] \tag{5}$$

Equation 5 was calculated across categories as in Equation 4.

We used the same approach for the analysis of multiple population samples. Let Ndn_{pop}, Cdn and Ncc_{pop}, Ccc be the number of populations, categories for *de novo* and case-control data respectively. The total Bayes Factor of a given gene was the product of Bayes Factors of all populations as in the main text, and all hyper parameters were estimated using Equation 2 in the main text.

The hyper-parameters $\phi_{1j} = (\gamma_{j(dn)}, \gamma_{j(cc)}, \beta_{j(dn)}, \beta_{j(cc)}, \rho_j, \nu_j)$ were estimated using a Hamiltonian Monte Carlo (HMC) Markov chain Monte Carlo (MCMC) method implemented in the `rstan` package (Carpenter et al., 2015; R Core Team, 2016). However, the model was first simplified by removing q (see below).

1.2.2.2 Simplified approximate case-control model

For case-control (transmitted) data, $q \sim Gamma(\rho, \nu)$, and hyper-parameters ρ and ν controlled the mean and dispersion of q; therefore, as in the previous studies (He et al., 2013; De Rubeis et al., 2014), ν was heuristically chosen (200 was used in all analyses) and $\frac{\rho}{\nu}$ = the mean frequency across genes in both cases and controls.

We simplified the case-control model by expressing it as

$$P(x_{ca}, x_{cn}|H_j) \quad = P(x_{ca}|x_{ca} + x_{cn}, H_j)P(x_{ca} + x_{cn}|H_j) \tag{6}$$

Because $x_{ca} \sim Pois(N_1 q \gamma_{cc})$ and $x_{cn} \sim Pois(N_0 q)$, assuming that x_{ca} and x_{cn} were **independent**, the case data could be modeled as:

$x_{ca}|x_{ca} + x_{cn}, H_j \sim Binomial(x_{ca} + x_{cn}, \theta|H_j)$

with $\theta|H_1 = \frac{N_1\gamma_{cc}}{N_1\gamma_{cc}+N_0}$ and $\theta|H_0 = \frac{N_1}{N_1+N_0}$

The marginal likelihood was

$P(x_{ca}|x_{ca} + x_{cn}, H_j) = \int P(x_{ca}|x_{ca} + x_{cn}, \gamma_{cc}, H_j)P(\gamma_{cc}|H_j)d\gamma_{cc}$

Based on simulation results, the first part $P(x_{ca}|x_{ca} + x_{cn}, H_j)$ can be used to infer mean RRs ($\bar{\gamma}_{cc}$); therefore only this part was used in the `extTADA` estimation process. However, to calculate Bayes Factors, we used full case-control models. We changed the order of integrals.

1.2.2.3 Control of an implied proportion of protective variants using the relative risk dispersion hyper-parameter

If $\bar{\gamma}$ and β were small then we could see a high proportion of protective variants when $\bar{\gamma}$ is not large. Although this might be of biological interest, it is

not currently accounted for in the model. To control the proportion of protective variants, we tested the relationship between β and $\bar{\gamma}$ in determining $\int Gamma(\bar{\gamma}_{dn}\beta_{dn}, \beta_{dn})$. We set this proportion very low (2%) (Figure S3) and built a nonlinear relationship $\beta = e^{a*\bar{\gamma}^b+c}$. The function *nls* in R was used to estimate a, b and c, as $6.77, -1.79$ and -0.22 respectively.

1.2.2.4 Calculate Bayes Factor for case/control data

At a given gene, Bayes Factor for each class was calculated as $BF = \frac{P(x_1,x_0|H_1)}{P(x_1,x_0|H_0)}$. The probability for each model ($H_j, j = 0, 1$) was calculated in order to rely only γ parameters as follows.

$$P(x_{ca}, x_{cn}|H_j) = P(x_{cn}|H_j)P(x_{ca}|x_{cn}, H_j) \tag{7}$$

- The first part $P(x_{cn}|H_j)$ was the same as De Rubeis et al. (2014):

$$P(x_{cn}|H_j) = \int P(x_{cn}|q, H_j)P(q|\rho, \nu, H_j)dq = NegBin(x_{cn}|\rho, \frac{N_0}{\nu + N_0}), j = 0, 1 \tag{8}$$

- The second part:

$$\begin{aligned} P(x_{ca}|H_j, x_{cn}) &= \int P(x_{ca}|q, \gamma_{cc})P(q|H_j, x_{cn})P(\gamma_{cc}|H_j)dqd\gamma_{cc} \\ &= \int [P(x_{ca}|q, \gamma_{cc})P(q|H_j, x_{cn})dq]\, P(\gamma_{cc}|H_j)d\gamma_{cc} \\ &= \int NegBin(x_{ca}|\rho + x_{cn}, \tfrac{N_0+\nu}{N_1\gamma_{cc}+N_0+\nu})P(\gamma_{cc}|H_j)d\gamma_{cc} \end{aligned} \tag{9}$$

To identify the lower and upper limits of γ_{CC} for the integral, we randomly sampled 10,000 times values from the $Gamma(\bar{\gamma}_{cc} * \beta_{cc}, \beta_{cc})$ and used the minimum and maximum values for the lower and upper limits respectively.

1.2.3 Infer parameters using MCMC results

The `rstan` package (Carpenter et al., 2015) was used to run MCMC processes. For simulation data, 5,000 times and a single chain were used. For real data, 20,000 times and three independent chains were used. In addition, for SCZ data we used two steps to obtain final results. Firstly, 10,000 times were run to obtain parameters. After that, we calculated β values from estimated mean RRs as the Equation described in Table S3. Finally, `extTADA` was re-run 20,000 times on the SCZ data with calculated β values set as constants to re-estimate mean RRs and the proportions of risk genes. For each MCMC process, a burning period = a half of total running times was used to assure that chains did not rely on their initial values. For example, we ran and removed 2,500 burning times before the 5,000 running times for simulation data.

We just chose 1,000 samples of each chain from MCMC results to do further analyses. For example, with a chain with 20,000 run times, the step to obtain a sample was 20 run times. For all estimated parameters from MCMC chains,

the convergence of each parameter was diagnosed using the estimated potential scale reduction statistic ($\hat{R}$) introduced in `Stan` (Carpenter et al., 2015). To produce heatmap plots, modes as well as the credible intervals (CIs) of estimated parameters, the Locfit (Loader, 2007) was used. The mode values were used as our estimated values for other calculations.

1.3 Supplementary Figures

This file includes Sup Figures below.

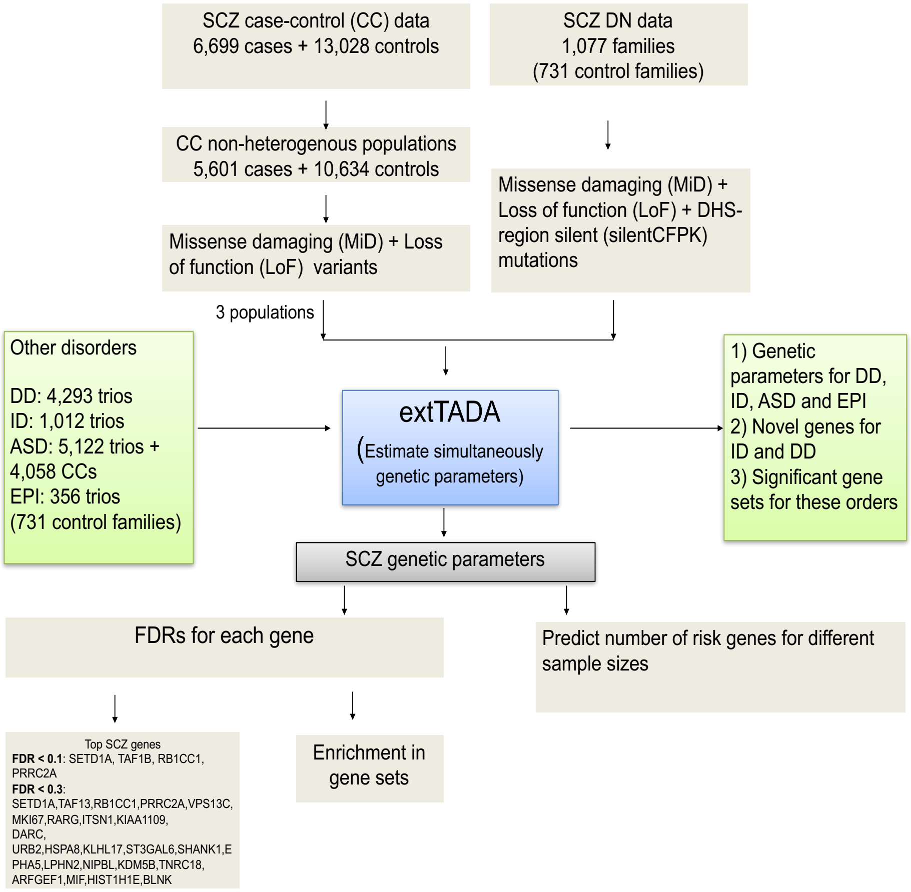

Figure S1: Workflow of data analysis.

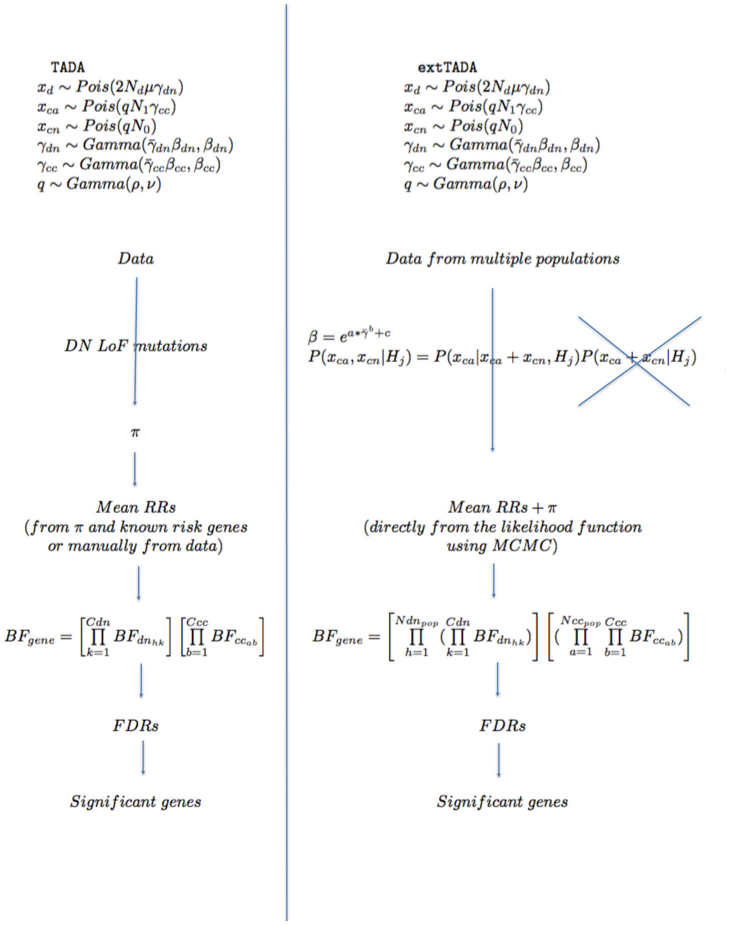

Figure S2: Comparison between `TADA` and `extTADA`. They both use the same model for *de novo* data (x_{dn} and case/control (x_{ca}, x_{cn}) data. `extTADA` combines all categories to obtain parameters and their credible intervals while `TADA` is based on LoF mutations. `extTADA` uses an approximate model for case-control data, and constrains β and $\bar{\gamma}$ in the estimation process. `extTADA` is designed to work for multiple populations. `TADA` can be used inside `extTADA`.

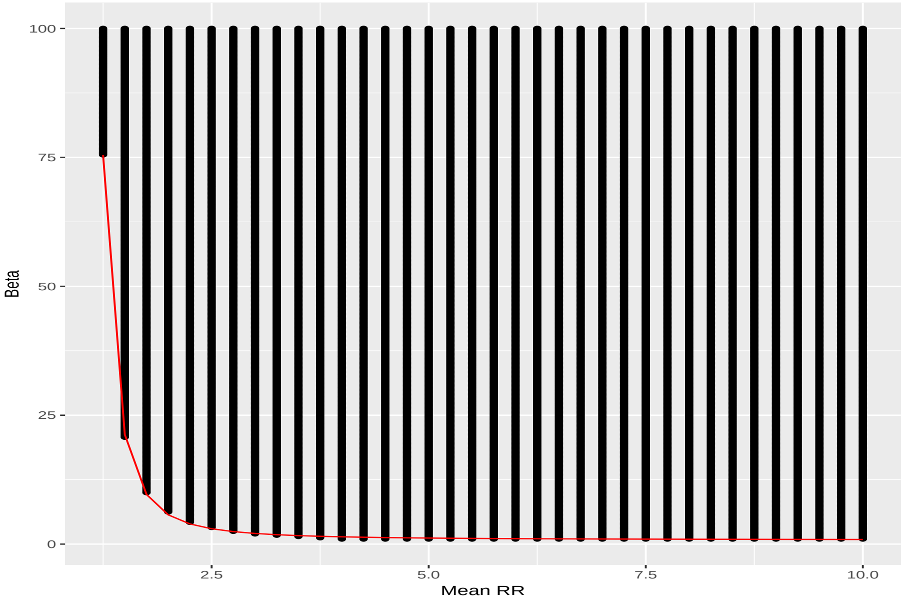

Figure S3: A grid of β and $\bar{\gamma}$ values. Points on the red line are corresponding with the proportion of protective variants less than 2%.

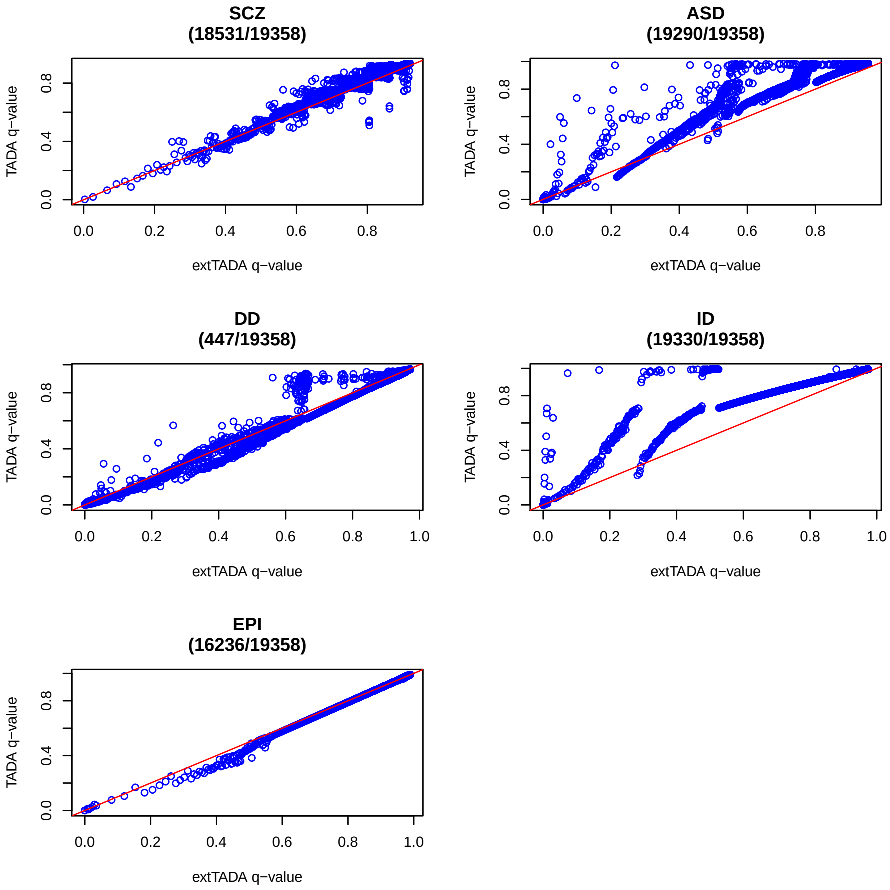

Figure S4: FDR q-values for all genes, for `extTADA` versus `TADA`. Panels (from top to bottom) show results for SCZ, ASD, DD, ID, and EPI.

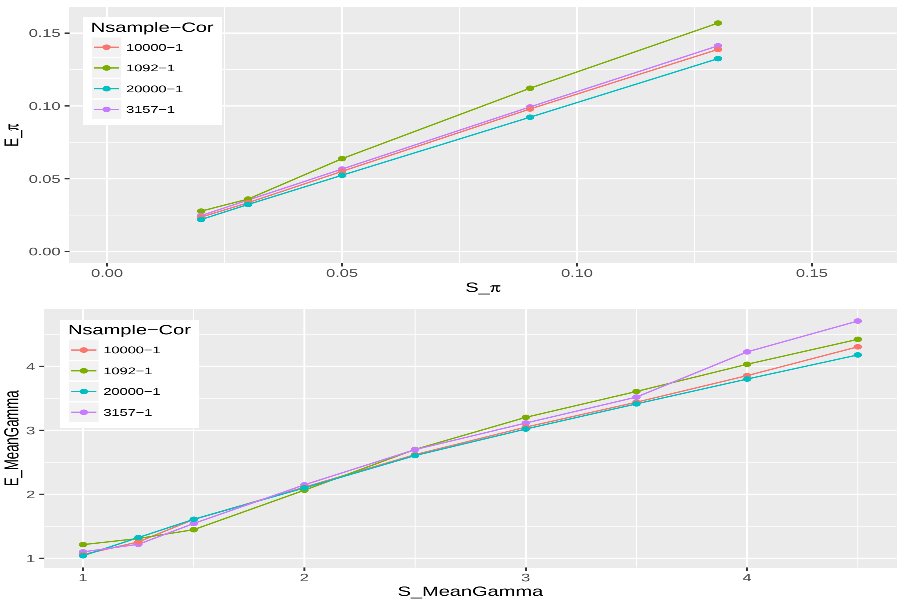

Figure S5: Correlations between estimated and simulated values for one CC class with different sample sizes. X and Y axes describe simulated (S) and estimated (E) values respectively. The top picture is for mean relative risks (MeanRRs) while the bottom picture is for the proportion of risk genes (π). Legends show sample sizes and correlations. These estimated values were averaged across simulation results. Detailed values are presented in Figure S6.

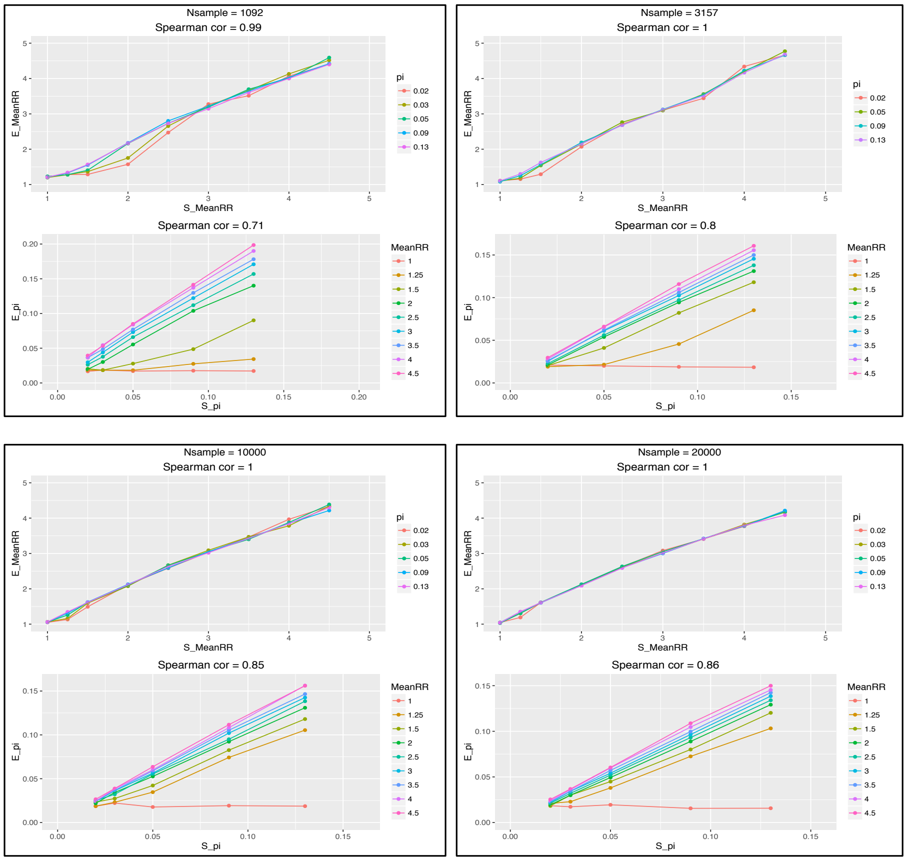

Figure S6: Correlation between simulated and estimated values for one-category case/control data.

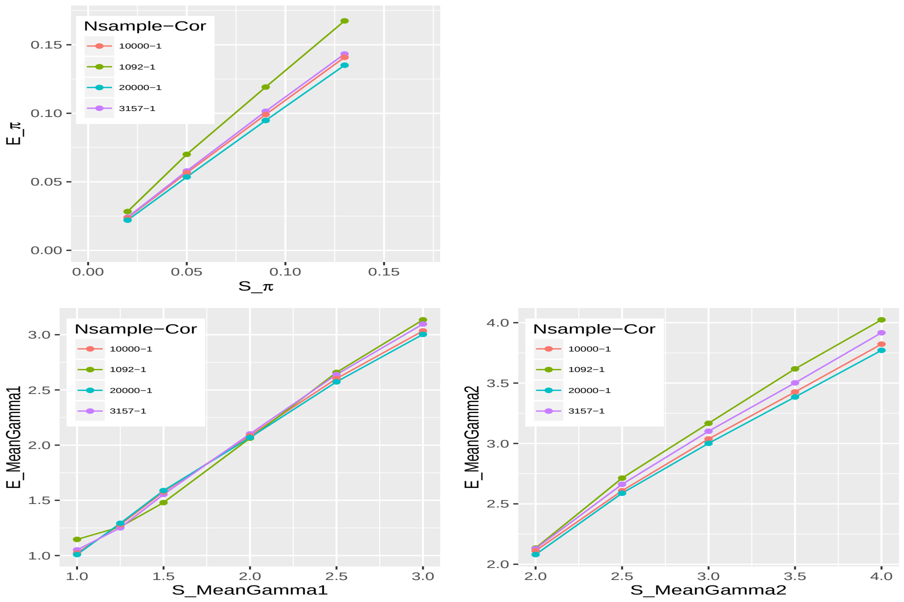

Figure S7: Correlations between estimated and simulated values for two CC class with different sample sizes. X and Y axes describe simulated (S) and estimated (E) values respectively. A range of mean relative risks for two classes (MeanGamma1 and MeanGamma2) and risk-gene proportions (π) were used in the simulation process. Legends show sample sizes and correlations. These estimated values were averaged across simulation results. Detailed values are presented in Figure S8.

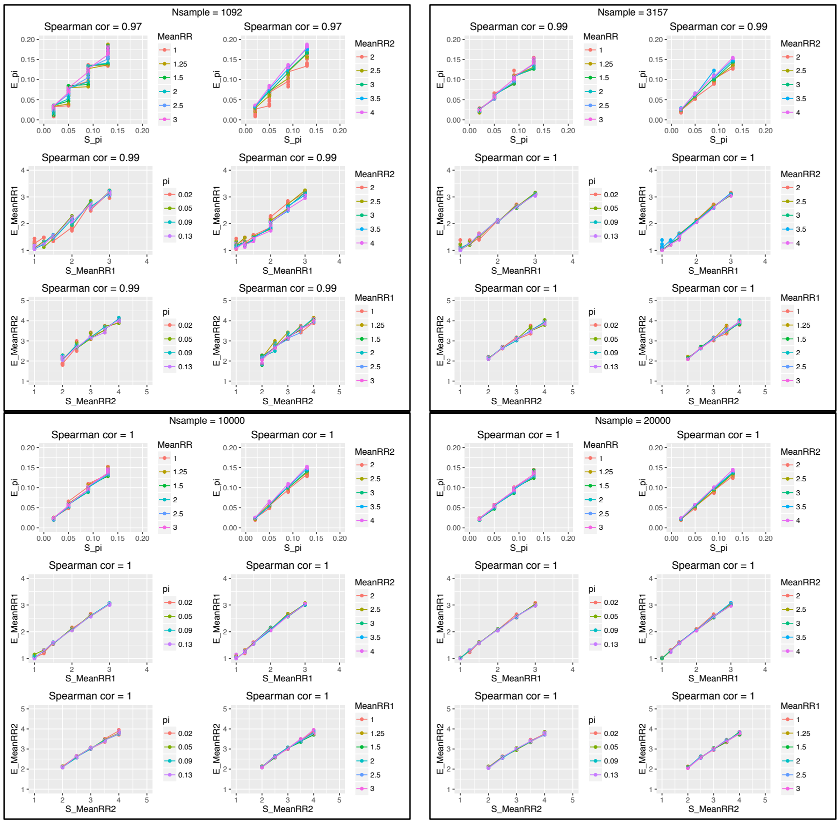

Figure S8: Correlation between simulated and estimated values for two-category case/control data.

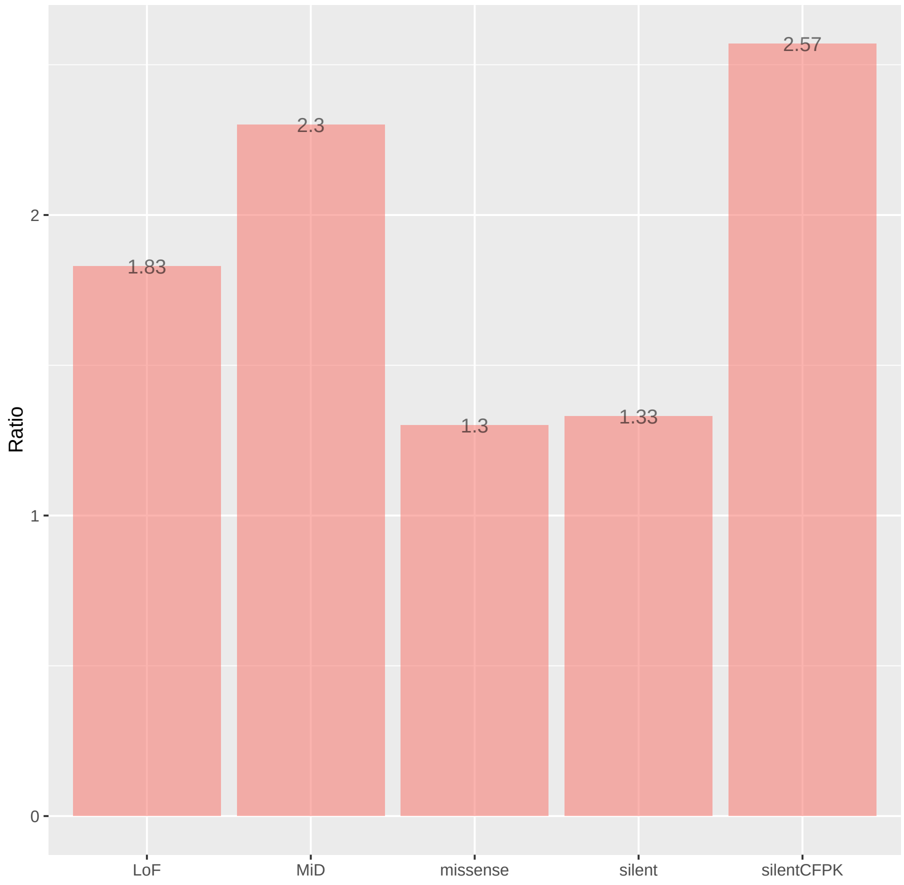

Figure S9: Ratios of *de novo* mutations between SCZ probands and controls (unaffected siblings). "silentFCPk" describes for silent mutations within frontal cortex-derived DHS (silentCerebrumfrontalocPk.narrowPeak). MiD mutations are missense mutations derived from 7 methods.

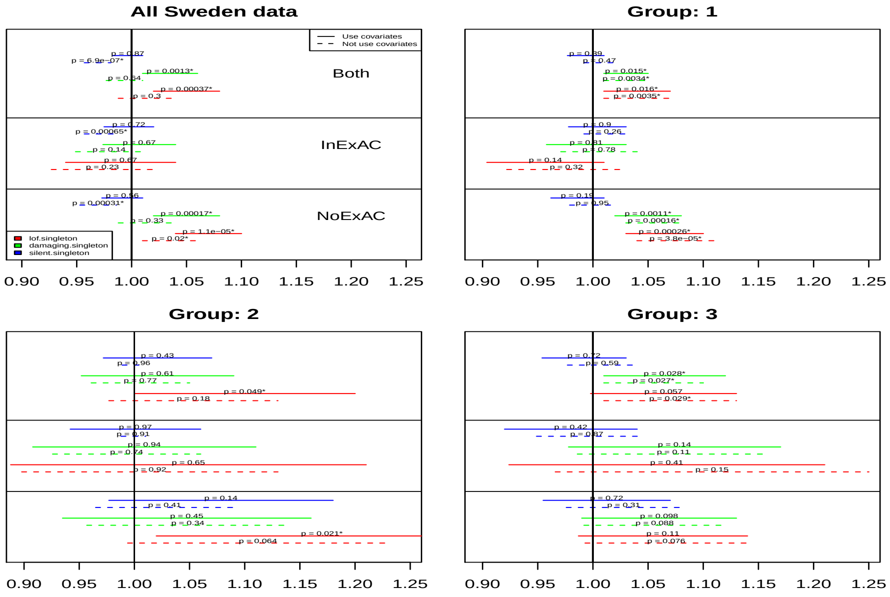

Figure S10: Odds ratios for the analysis of all case-control samples. Top left picture shows odds ratios for all Sweden samples while the three other pictures show odds ratios for three groups after the clustering process. Only group 1 and 3 are used in the current analysis because there are strong differences between results using covariates and not using covariates in group 2. P values were calculated for variants in (InExAC), not in (NoExAC) the ExAC database, and all variants (Both).

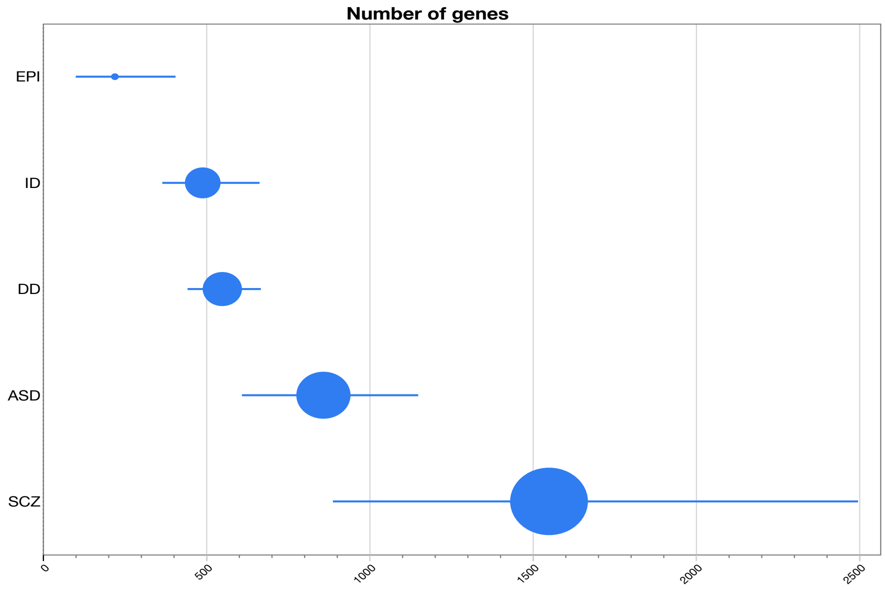

Figure S11: Estimated gene counts for all disorders, with 95% CIs. Point sizes are proportional to sample sizes.

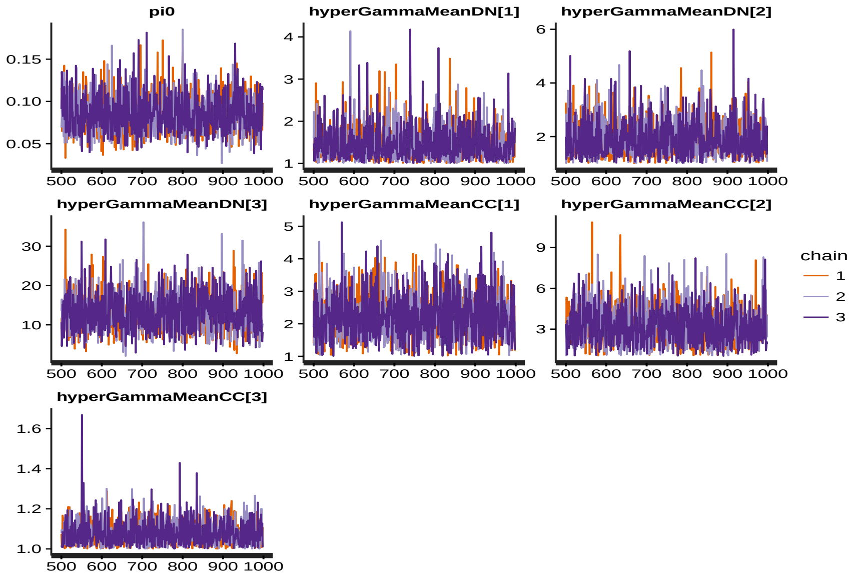

Figure S12: MCMC results for SCZ data. These are obtained after 20,000 iterations.

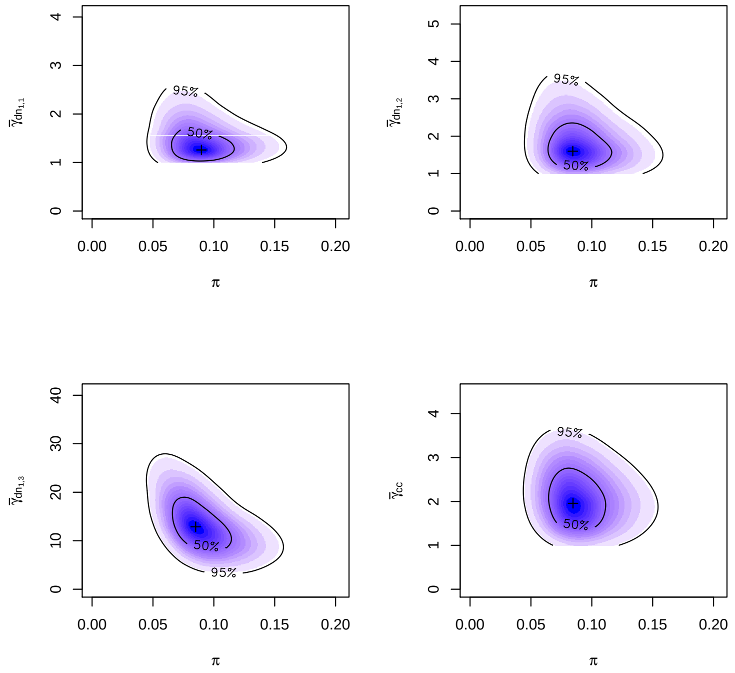

Figure S13: SCZ genetic parameters when mean RRs of case-control data are equal.

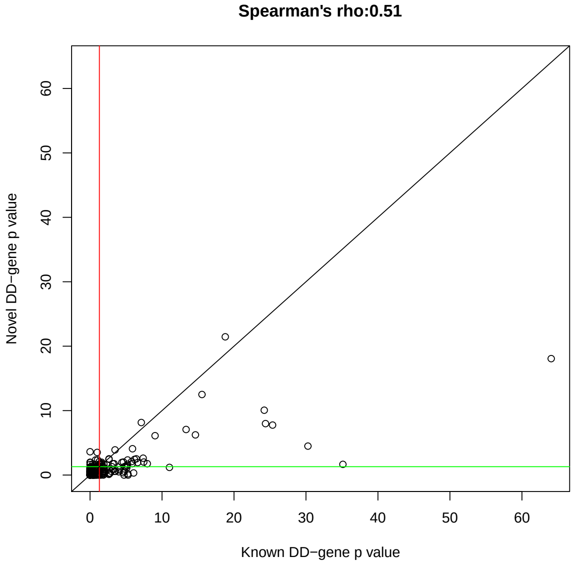

Figure S14: The correlation of gene-set p values (values are -log10(p values)) between known and novel genes from `extTADA` results for DD

.

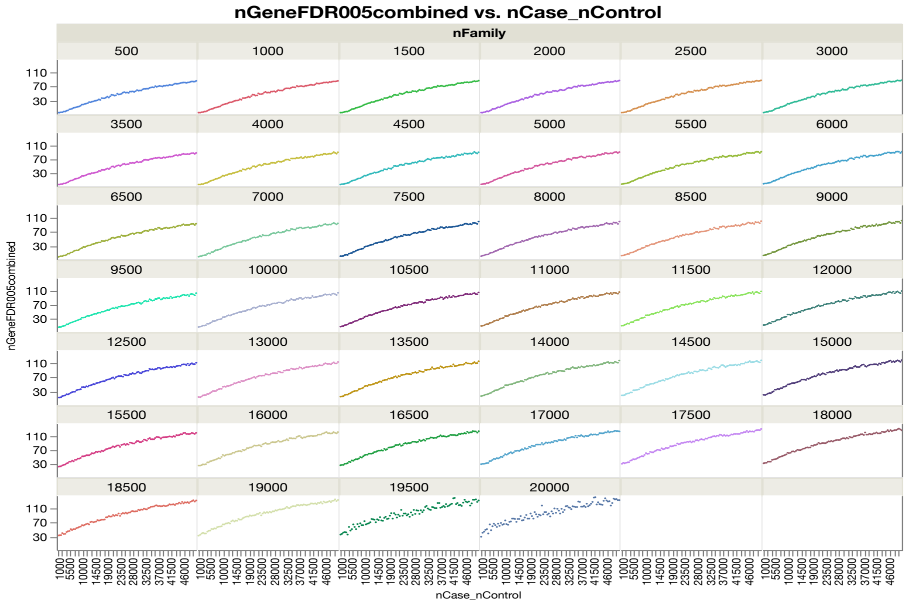

Figure S15: Number of risk genes with different sample sizes based on genetic architecture predicted by `extTADA`. Case/control number is only for cases (with equal controls) (x-axis); each panel shows results for a given number of trios.

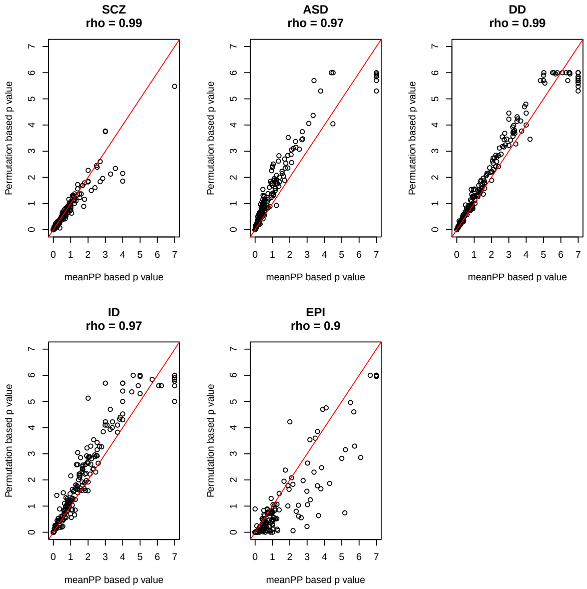

Figure S16: The correlation of gene-set p values (-log(p value)) between mean posterior profitability (meanPP) based method and permutation based methods.

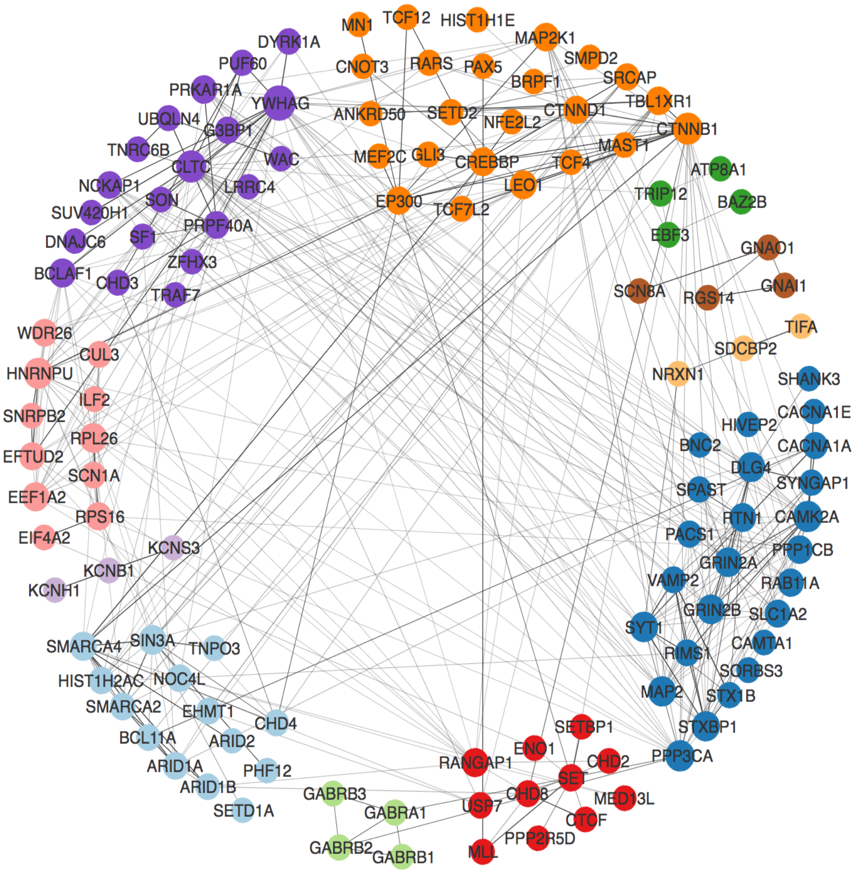

Figure S17: GeNets InWeb PPI network for 288 NDD genes, with direct edges only.

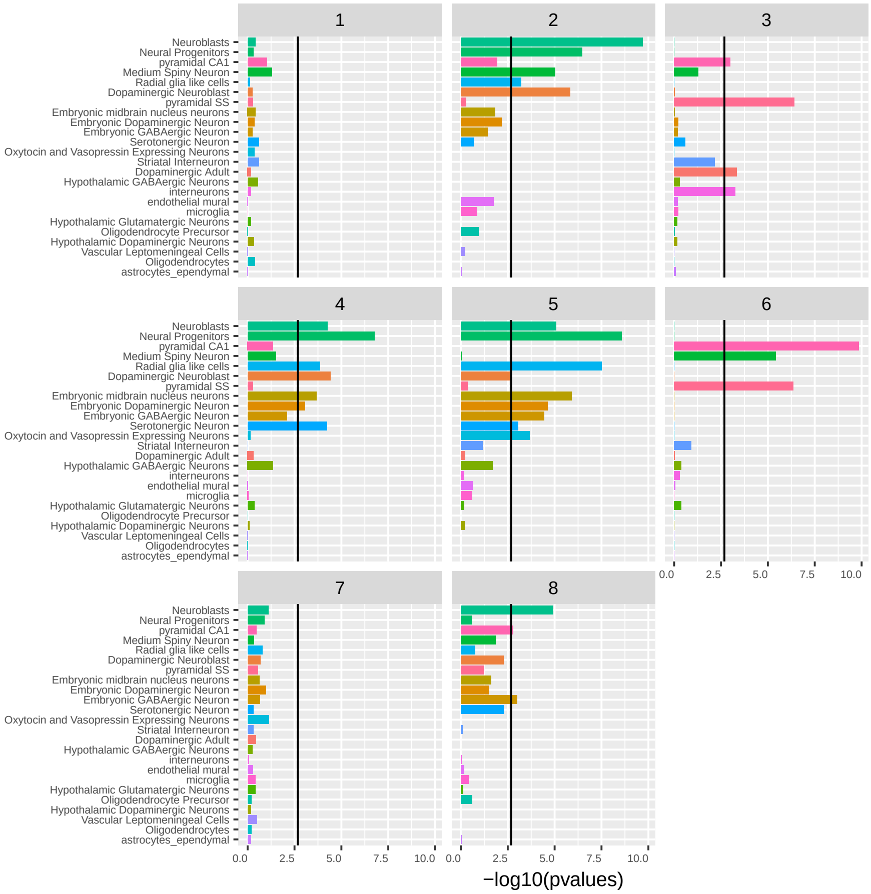

Figure S18: Evaluation of enrichment of each community from GeNets results in brain scRNAseq datasets from mouse.

1.4 Supplementary Tables

This part includes Sup Tables below.

Source	Disease	DN	DN control	Case	Control
Fromer et al. (2014)	SCZ	617			
Girard et al. (2011)	SCZ	14			
Gulsuner et al. (2013)	SCZ	105	84		
McCarthy et al. (2014)	SCZ	57			
Xu et al. (2012)	SCZ	231	34		
Guipponi et al. (2014)	SCZ	53			
Genovese et al. (2016)	SCZ			4954/4248	6239/5865
Singh et al. (2016)	SCZ			1745/1353	6789/4769
Deciphering Developmental Disorders Study (2017)	DD	4293			
EuroEPINOMICS-RES Consortium et al. (2014)	EPI	356			
De Ligt et al. (2012)	ID	100			
Hamdan et al. (2014)	ID	41			
Rauch et al. (2012)	ID	51	20		
Lelieveld et al. (2016)	ID	820			
Turner et al. (2016)	ASD	5122			
De Rubeis et al. (2014)	ASD			404	3654
Iossifov et al. (2012)	ASD		343		
ORoak et al. (2012)	ASD		50		
Sanders et al. (2012)	ASD		200		

Table S1: *de novo* and case/control data. For ASD studies, Turner et al. (2016) integrated previous results in their study; therefore only *de novo* meta data in their study are shown in the table. In addition, for ASD case-control data, only one homogeneous Sweden population from De Rubeis et al. (2014) was used. For case-control data of SCZ, after correcting for the population stratification, only 4,248 cases (3,157 + 1,091) + 5,865 (4,672 + 1,193) controls from Genovese et al. (2016) and 1,353 cases + 4,769 controls from Singh et al. (2016) are used in this study.

Gene set name	Abbreviation	Author
Missense constrained genes	constrained	Samocha et al. (2014)
Loss-of-function tolerance genes	pLI90	Lek et al. (2015)
RBFOX2 and RBFOX1/3 genes	rbfox2, rbfox13	Weyn-Vanhentenryck et al. (2014)
FMRP genes	fmrp	Darnell et al. (2011)
CELF4 genes	celf4	Wagnon et al. (2012)
synaptic genes	synaptome	Pirooznia et al. (2012)
microRNA-137	mir137	Robinson et al. (2015)
PSD-95 complex genes	psd95	Bayés et al. (2011)
ARC and NMDA receptors genes	nmdarc	Kirov et al. (2012)
Essential genes	essential	Ji et al. (2016)
Human accelerated regions and primate accelerated regions	HARs, PARS	Lindblad-Toh et al. (2011)
Known ID gene sets	IDallKnownGenes	Lelieveld et al. (2016)
Voltage-gated Calcium Channel Genes	vacc	
CHD8 promoter targets	chd8 hNSC, chd8 hNSC specific, chd8 human brain, chd8 hNSC human brain, chd8 hNSC human mouse	Cotney et al. (2015)
Allelic-biased expression genes in neurons	AlleleBiasedExpression.Neuron	Lin et al. (2012)
24 gene sets from 24 modules	Module.M1..M24	Johnson et al. (2016)
de novo copy number variants		Genovese et al. (2016)
ASD	CNV.denovo.gain/loss.asd	
Bipolar	CNV.denovo.gain/loss.bd	
SCZ	CNV.denovo.gain/loss.scz	
MiD and LoF *de novo* mutations		
DD	DD.allDenovoMiDandLoF	
ASD	ASD.allDenovoMiDandLoF	
EPI	EPI.allDenovoMiDandLoF	
ID	ID.allDenovoMiDandLoF	

Table S2: Abbreviations of known gene sets used in this study.

Data model	*Parameter prior*	*Hyper prior*
$x_{dn} \sim P(2N_{dn}\mu\gamma_{dn})$	$\gamma_{dn} \sim Gamma(\bar{\gamma}_{dn} * \beta_{dn}, \beta_{dn})$<br>$\beta_{dn} = e^{a*\bar{\gamma}_{dn}^{b}+c}$	$\bar{\gamma}_{dn} \sim Gamma(\bar{\bar{\gamma}}_{dn}, \bar{\beta}_{dn})$
$x_{ca} \sim P(N_1 q\gamma_{cc})$	$\gamma_{cc} \sim Gamma(\bar{\gamma}_{cc} * \beta_{cc}, \beta_{cc})$<br>$\beta_{cc} = e^{a*\bar{\gamma}_{cc}^{b}+c}$<br>$q \sim Gamma(\rho, \nu)$	$\bar{\gamma}_{cc} \sim Gamma(\bar{\bar{\gamma}}_{cc}, \bar{\beta}_{cc})$<br>$\frac{\rho}{\nu} = mean(\sum(x_{cn} + x_{ca}))$<br>$\nu = 200$
$x_{cn} \sim P(N_0 q)$	$q \sim Gamma(\rho, \nu)$	$\frac{\rho}{\nu} = mean(\sum(x_{cn} + x_{ca}))$<br>$\nu = 200$
	$\pi \sim \ Beta(1, 5)$	

Table S3: Parameter information used in all analyses. N_{dn}, N_1, N_0 are sample sizes of families, cases and controls respectively. $\bar{\gamma}$ is mean RRs and β controls the dispersion of γ. $\bar{\bar{\gamma}}$ and $\bar{\beta}$ are priors for $\bar{\gamma}$ and are set in advance (they are inferred from simulation data). β is inferred from the equation $e^{a*\bar{\gamma}^{b}+c}$ inside the estimation process with a = 6.83, b = -1.29 and c = -0.58.

Disease	$\hat{\pi}$(%)	dnLoF $\hat{\bar{\gamma}}$	dnMiD $\hat{\bar{\gamma}}$	dnCPPk $\hat{\bar{\gamma}}$	CC1 $\hat{\bar{\gamma}}$	CC2 $\hat{\bar{\gamma}}$	CC3 $\hat{\bar{\gamma}}$
SCZ	6.7	16.2	-0.2	-5.3	2.0	2.0	-0.2
DD	3.6	102.1	36.7				
ID	0.8	409.8	140.6				
EPI	1.0	169.3	81.4				
ASD	1.8	74.8	8.7		7.5		

Table S4: Estimated genetic parameters from `TADA` for *de novo* (dn) and case/-control (CC) data. These parameters were estimated directly from variant counts without using prior information or known gene sets. LoF de novo mutations were used to estimate risk-gene proportions. Negative results are because of unconvergent results.

Parameter		Q50	Q5	Q95
π	0.02	0.0224	0.0125	0.0253
	0.05	0.0535	0.0351	0.0611
	0.09	0.0965	0.0752	0.1063
	0.13	0.1381	0.11	0.149
$\bar{\gamma}_{DN}$	5	4.265	3.5608	4.947
	10	8.575	5.7255	10.4417
	15	13.23	9.9955	15.925
	20	17.07	14.2005	20.3087
$\bar{\gamma}_{CC}$	1.5	1.64	1.5938	1.7888
	2	2.21	2.1638	2.2662
	2.5	2.76	2.7138	2.8575
	3	3.225	3.14	3.31
	3.5	3.675	3.5812	3.7663

Table S5: Simulated and estimated values of *de novo* (DN) and case-control (CC) parameters. Q50, Q5 and Q95 are for quantile values of 0.5, 0.05 and 0.95 respectively.

pi	dn_RR	cc_RR	e.pi Q50	Q5	Q95	e.dn_RR Q50	Q5	Q95	e.cc_RR Q50	Q5	Q95
0.02	5	1.5	0.0126	0.0051	0.0224	4.72	2.65	9.22	1.83	1.70	2.53
0.02	5	2	0.0158	0.0054	0.0394	4.08	2.33	11.88	2.27	1.88	3.01
0.02	5	2.5	0.0230	0.0093	0.0419	3.49	2.05	10.25	2.83	2.22	3.43
0.02	5	3	0.0219	0.0123	0.0355	3.52	2.06	8.49	3.31	2.74	4.25
0.02	5	3.5	0.0269	0.0171	0.0373	3.76	2.10	9.59	3.80	3.15	4.73
0.02	10	1.5	0.0185	0.0085	0.0280	5.72	2.82	10.08	1.80	1.54	2.57
0.02	10	2	0.0176	0.0076	0.0373	5.22	2.55	13.43	2.24	1.85	3.05
0.02	10	2.5	0.0218	0.0118	0.0342	5.13	2.39	17.29	2.80	2.27	3.57
0.02	10	3	0.0227	0.0137	0.0344	7.29	2.35	15.88	3.28	2.64	4.23
0.02	10	3.5	0.0255	0.0163	0.0328	7.87	2.79	14.28	3.73	3.07	4.69
0.02	15	1.5	0.0152	0.0046	0.0315	11.63	4.65	28.84	1.77	1.48	2.67
0.02	15	2	0.0213	0.0091	0.0348	10.19	3.32	23.63	2.25	1.86	2.84
0.02	15	2.5	0.0230	0.0118	0.0377	9.73	4.00	20.84	2.69	2.06	3.50
0.02	15	3	0.0226	0.0128	0.0370	10.51	3.53	21.52	3.18	2.55	4.11
0.02	15	3.5	0.0240	0.0140	0.0364	10.56	3.58	20.62	3.62	3.04	4.70
0.02	20	1.5	0.0138	0.0062	0.0398	14.96	5.47	47.26	1.68	1.35	2.29
0.02	20	2	0.0188	0.0079	0.0363	14.10	4.79	36.13	2.28	1.81	3.20
0.02	20	2.5	0.0233	0.0110	0.0343	13.61	5.67	26.36	2.76	2.12	3.54
0.02	20	3	0.0243	0.0140	0.0371	13.89	6.44	23.95	3.41	2.63	4.30
0.02	20	3.5	0.0240	0.0146	0.0352	14.87	6.90	24.81	3.77	2.96	4.65
0.05	5	1.5	0.0343	0.0120	0.0688	4.55	2.26	14.06	1.71	1.51	2.17
0.05	5	2	0.0479	0.0279	0.0699	4.86	2.32	10.44	2.21	1.88	2.60
0.05	5	2.5	0.0556	0.0351	0.0743	4.59	2.06	8.03	2.81	2.31	3.17
0.05	5	3	0.0558	0.0427	0.0722	4.36	2.08	8.18	3.35	2.91	3.74
0.05	5	3.5	0.0621	0.0435	0.0727	3.65	1.89	7.36	3.73	3.22	4.48
0.05	10	1.5	0.0381	0.0161	0.0723	9.20	3.92	15.41	1.74	1.45	2.18
0.05	10	2	0.0531	0.0293	0.0801	8.71	3.70	12.99	2.26	1.91	2.71
0.05	10	2.5	0.0528	0.0386	0.0727	8.76	4.15	14.48	2.74	2.47	3.11
0.05	10	3	0.0569	0.0416	0.0737	8.22	4.47	13.57	3.25	2.83	3.72
0.05	10	3.5	0.0615	0.0491	0.0733	8.06	3.97	13.17	3.66	3.30	4.29
0.05	15	1.5	0.0406	0.0182	0.0877	13.51	6.94	24.71	1.67	1.43	1.98
0.05	15	2	0.0489	0.0311	0.0723	14.04	8.16	22.70	2.19	1.90	2.61
0.05	15	2.5	0.0522	0.0327	0.0734	13.13	8.28	20.66	2.72	2.38	3.11
0.05	15	3	0.0577	0.0449	0.0732	12.37	7.27	18.59	3.19	2.83	3.75
0.05	15	3.5	0.0607	0.0465	0.0756	11.97	8.23	18.55	3.61	3.11	4.29
0.05	20	1.5	0.0418	0.0205	0.0814	18.37	9.74	32.56	1.63	1.37	1.97
0.05	20	2	0.0482	0.0325	0.0697	17.08	10.14	29.26	2.27	1.91	2.60
0.05	20	2.5	0.0537	0.0406	0.0733	16.59	10.57	23.23	2.77	2.29	3.06
0.05	20	3	0.0569	0.0424	0.0770	16.15	10.37	24.32	3.23	2.84	3.75
0.05	20	3.5	0.0596	0.0449	0.0765	15.50	10.23	21.45	3.75	3.19	4.61
0.09	5	1.5	0.0767	0.0404	0.1207	4.46	2.17	9.59	1.66	1.51	1.97
0.09	5	2	0.0904	0.0666	0.1115	4.52	2.04	7.33	2.23	2.03	2.54
0.09	5	2.5	0.0963	0.0753	0.1256	4.70	2.52	7.54	2.79	2.49	3.11
0.09	5	3	0.1040	0.0879	0.1217	3.90	2.08	6.71	3.19	2.85	3.68
0.09	5	3.5	0.1039	0.0876	0.1211	4.22	2.34	7.93	3.70	3.35	4.13
0.09	10	1.5	0.0778	0.0423	0.1208	10.01	5.56	17.73	1.64	1.46	1.93
0.09	10	2	0.0925	0.0660	0.1196	9.26	5.85	13.45	2.16	1.96	2.49
0.09	10	2.5	0.0963	0.0729	0.1170	9.30	7.16	12.50	2.82	2.40	3.18
0.09	10	3	0.0992	0.0831	0.1189	9.25	6.11	12.76	3.22	2.95	3.61
0.09	10	3.5	0.1070	0.0885	0.1222	8.29	5.81	10.94	3.67	3.36	4.20
0.09	15	1.5	0.0822	0.0507	0.1257	14.59	9.22	22.62	1.61	1.43	1.89
0.09	15	2	0.0911	0.0668	0.1217	14.35	9.39	20.13	2.16	1.94	2.45
0.09	15	2.5	0.0978	0.0754	0.1202	13.77	10.40	17.99	2.72	2.40	3.00
0.09	15	3	0.0997	0.0844	0.1206	13.50	10.60	16.88	3.13	2.82	3.49
0.09	15	3.5	0.1036	0.0861	0.1229	12.95	9.89	16.86	3.60	3.20	4.15
0.09	20	1.5	0.0804	0.0495	0.1236	19.92	13.06	31.58	1.60	1.38	1.82
0.09	20	2	0.0920	0.0694	0.1205	18.18	12.71	24.69	2.21	1.95	2.51
0.09	20	2.5	0.0958	0.0742	0.1166	18.28	13.76	22.90	2.75	2.49	3.05
0.09	20	3	0.0974	0.0816	0.1202	17.55	13.32	22.38	3.28	2.95	3.59
0.09	20	3.5	0.1067	0.0925	0.1171	16.49	13.66	20.83	3.68	3.32	4.21
0.13	5	1.5	0.1163	0.0720	0.1671	4.87	2.51	8.11	1.65	1.49	1.83
0.13	5	2	0.1250	0.0991	0.1603	5.15	2.82	7.72	2.22	2.04	2.53
0.13	5	2.5	0.1387	0.1173	0.1654	4.65	2.51	7.04	2.77	2.52	3.08
0.13	5	3	0.1469	0.1220	0.1649	4.40	2.83	6.17	3.20	2.93	3.47
0.13	5	3.5	0.1467	0.1293	0.1747	4.45	2.46	6.13	3.69	3.36	4.25
0.13	10	1.5	0.1094	0.0707	0.1660	10.69	7.35	17.96	1.68	1.53	1.84
0.13	10	2	0.1306	0.1113	0.1529	9.40	6.89	13.22	2.17	2.01	2.36
0.13	10	2.5	0.1432	0.1197	0.1595	9.15	7.21	11.97	2.73	2.52	3.01
0.13	10	3	0.1457	0.1308	0.1682	8.89	6.64	11.08	3.23	3.00	3.54
0.13	10	3.5	0.1497	0.1320	0.1728	8.54	6.62	10.61	3.60	3.26	3.97
0.13	15	1.5	0.1180	0.0778	0.1677	15.08	10.41	22.63	1.60	1.46	1.80
0.13	15	2	0.1277	0.1044	0.1593	14.45	11.56	18.45	2.15	1.96	2.40
0.13	15	2.5	0.1380	0.1124	0.1625	14.34	11.23	17.93	2.72	2.45	3.04
0.13	15	3	0.1432	0.1254	0.1667	13.41	11.03	16.81	3.13	2.87	3.63
0.13	15	3.5	0.1488	0.1281	0.1674	13.00	10.35	16.30	3.56	3.20	3.98
0.13	20	1.5	0.1203	0.0862	0.1765	19.72	13.93	26.77	1.61	1.48	1.83
0.13	20	2	0.1325	0.1093	0.1546	18.54	15.11	23.43	2.21	1.99	2.39
0.13	20	2.5	0.1351	0.1130	0.1601	18.38	14.63	22.97	2.79	2.50	3.00
0.13	20	3	0.1434	0.1256	0.1645	18.43	14.94	22.27	3.24	2.94	3.60
0.13	20	3.5	0.1488	0.1320	0.1637	16.81	13.99	20.18	3.64	3.34	4.11

Table S6: Estimated values for the cases in Table S5, for each unique set of parameter values. The first three columns are simulated values. The following columns show estimated π, *de novo* mean relative risk (dn RR) and case-contorl (cc) RR; for each parameter, shown are median (Q50) and 5th and 95th %-iles (Q5 and Q95) estimates over 100 simulation replicates.

$\bar{\beta}_{DN}$	$\bar{\beta}_{CC}$	e.π	e.$\bar{\beta}_{DN}$	e.$\bar{\beta}_{CC}$	e.β_{DN}	e.β_{CC}	FDR0.01	FDR0.05	FDR0.1	FDR0.25	FDR0.5
0.01	0.11	0.0008985	12.16	2.37	0.82	1.38	0	0	0	0	0
0.01	0.14	0.0013925	7.76	2.02	0.84	2.08	0	0	0	0	0
0.01	0.2	0.0011444	7.38	1.66	0.86	3.2	0	0	0	0	0
0.01	0.33	0.0014319	10.32	1.46	0.83	5.06	0	0	0	0	0
0.01	1	0.0010192	6.12	1.26	0.87	24.04	0	0	0	0	0
0.02	0.11	0.0012389	5.7	1.72	0.88	2.07	0	0	0	0	0
0.02	0.14	0.00339	6.25	1.6	0.88	5.1	0	0	0	0	0
0.02	0.2	0.0036757	12.62	1.53	0.83	4.77	0	0	0	0	0
0.02	0.33	0.0040126	3.34	1.32	1.14	15.47	0	0	0	0	0
0.02	1	0.0057346	5.27	1.15	0.92	51.7	0	0	0	0	0
0.03	0.11	0.0012311	7.23	1.63	0.87	2.43	0	0	0	0	0
0.03	0.14	0.0009967	6.37	1.61	0.87	3.88	0	0	0	0	0
0.03	0.2	0.0022818	5.16	1.55	0.92	5.4	0	0	0	0	0
0.03	0.33	0.0110319	4.16	1.35	1.02	16.06	0	0	0	0	2
0.03	1	0.004111	3.75	1.19	1.03	42.34	0	0	0	0	0
0.05	0.11	0.0018204	5.78	1.38	0.9	5.92	0	0	0	0	0
0.05	0.14	0.0015779	7.84	2.04	0.86	2.14	0	0	0	0	0
0.05	0.2	0.0034645	4.75	1.34	0.94	9.15	0	0	0	0	0
0.05	0.33	0.0123621	1.75	1.24	2.27	24.09	0	0	0	0	0
0.05	1	0.0035687	3.63	1.18	1.03	47.33	0	0	0	0	0

Table S7: Estimated values in the case $\pi = 0$ and $\bar{\gamma} = 1$. The first two columns are $\bar{\beta}$ values (prior information of $\bar{\gamma}$: $\bar{\gamma} \sim Gamma(1, \bar{\beta})$). The third to the seventh columns are genetic parameters estimated from `extTADA`. Next columns are the number of risk genes estimated with the corresponding FDR values in the header.

Disease	Mutation	Count	Sample size	Mutation count per sample size
SCZ	silentFCPk	50	1077	0.05
	MiD	105	1077	0.1
	LoF	116	1077	0.11
ASD	MiD	618	5122	0.12
	LoF	638	5122	0.12
ID	MiD	223	1022	0.22
	LoF	225	1022	0.23
EPI	MiD	69	356	0.19
	LoF	52	356	0.15
DD	MiD	1041	4293	0.24
	LoF	1066	4293	0.25

Table S8: *de novo* mutation counts of categories and their mutation counts per sample size for schizophrenia (SCZ), autism spectrum disorder (ASD), epilepsy (EPI), intellectual disorder (ID) and developmental disorder (DD).

Parameters	Estimated mode	lCI	uCI
SCZ_pi_silentFCPkdn	0.6	0	19.8
SCZ_hyperGammaMean_silentFCPkdn	1.5802	1.001	21.5139
SCZ_pi_MiDdn	1.2	0	23.7
SCZ_hyperGammaMean_MiDdn	1.7486	1	17.8548
SCZ_pi_LoFdn	5.48	1.24	20.62
SCZ_hyperGammaMean_LoFdn	11.1857	3.3973	31.3602
SCZ_pi_MiD+LoFcc	6.9	2.96	13.6
SCZ_hyperGammaMean_MiD+LoFcc	2.0176	1.2133	5.3694
SCZ_hyperGammaMean_MiD+LoFcc	3.2288	1.2372	17.1478
SCZ_hyperGammaMean_MiD+LoFcc	1.0691	1.0002	2.9574

Table S9: Genetic parameters for SCZ data if single class is used in the analysis.

Parameters	Estimated mode	lCI	uCI
SCZ_pi0	9.37	5.47	15.12
SCZ_meanRR_silentFCPkdenovo	1.3068	1.0005	2.7489
SCZ_meanRR_MiDdenovo	2.2246	1.0006	5.3491
SCZ_meanRR_LoFdenovo	15.1491	5.8606	27.3941
SCZ_meanRR_MiD+LoFccPop1	1.8677	1.0374	3.0736
SCZ_meanRR_MiD+LoFccPop2	2.2632	1.003	4.9168
SCZ_meanRR_MiD+LoFccPop3	1.0372	1.0002	1.1807
ASD_pi	9.47	7.61	12.27
ASD_meanRR_MiDdenovo	5.09	2.47	10.51
ASD_meanRR_LoFdenovo	20.23	12.21	32.31
ASD_meanRR_LoFcc	2.48	1.48	5.95
ID_pi	3.53	2.63	4.56
ID_meanRR_MiDdenovo	35.29	21.46	51.62
ID_meanRR_LoFdenovo	105.44	74.58	143.02
DD_pi	1.91	1.57	2.37
DD_meanRR_MiDdenovo	22.72	13.91	34.36
DD_meanRR_LoFdenovo	99.94	75.39	127.18
EPI_pi	1.67	0.96	3.1
EPI_meanRR_MiDdenovo	71.77	37.15	125.14
EPI_meanRR_LoFdenovo	94.98	51.73	176.16

Table S10: SCZ and NDD genetic parameters after adjusting mutation rates.

Parameter	Mode	lCI	uCI
pi0	0.0821	0.0487	0.1398
hyperGammaMeanDN[1]	1.2199	1.0001	2.2
hyperGammaMeanDN[2]	1.4407	1.0043	2.9893
hyperGammaMeanDN[3]	11.9591	4.1894	23.9414
hyperGammaMeanCC	1.9498	1.0845	3.2072

Table S11: Estimated genetic parameters for SCZ data with the same mean RRs for case-control data.

Parameters	Estimated mode	lCI	uCI
SCZ_pi	0.0732	0.0306	0.1506
SCZ_meanRR_silentFCPkdenovo	1.2353	1.0021	3.6086
SCZ_meanRR_MiDdenovo	1.4459	1.0008	4.7004
SCZ_meanRR_LoFdenovo	12.0403	4.6136	25.8786
SCZ_meanRR_MiD+LoFccPop1	1.5856	1.1255	4.0881
SCZ_meanRR_MiD+LoFccPop2	1.7361	1.0438	4.8856
SCZ_meanRR_MiD+LoFccPop3	1.0698	1.0001	2.9991

Table S12: SCZ genetic parameters using all variants in and not in ExAC database (InExAC + NoExAC).

Table S13: extTADA results of SCZ risk gene identification (See LongSupTables.xlsx Download).

Table S14: extTADA results of SCZ risk gene identification after adjusting mutation rates (See LongSupTables.xlsx Download).

Table S15: extTADA risk gene identification results of ID data (See LongSupTables.xlsx Download).

Table S16: extTADA risk gene identification results of DD data (See LongSupTables.xlsx Download).

Table S17: extTADA risk gene identification results of ASD data (See LongSupTables.xlsx Download).

Table S18: extTADA risk gene identification results of EPI data (See LongSupTables.xlsx Download).

Disorder	FDR threshold	m_0	mean(m_1)	sd(m_1)	Unadjusted P value	FDR
DD	0.05	3406.4	3202.7	305.4	0.19	0.57
ID	0.05	4017.6	3442.6	351.9	0.057	0.25
EPI	0.05	4093.3	4454.4	3681.9	0.28	0.67
ASD	0.05	4688.8	3492	632.4	0.049	0.25
DD	0.1	3123.8	3203	271.8	0.56	0.8
ID	0.1	3481.4	3442.4	308.2	0.44	0.75
EPI	0.1	3787	4445.1	3460.5	0.36	0.72
ASD	0.1	4413.2	3488.2	546.9	0.063	0.25
DD	0.3	2464.2	3202.9	182	1	1
ID	0.3	2997.3	3442.2	157.6	1	1
EPI	0.3	2540.8	4448.7	2393.6	0.92	1
ASD	0.3	3358.3	3489.7	348.6	0.6	0.8

Table S19: Test correlation between detected genes with different FDR thresholds and gene sizes. The first and second columns show neurodevelopmental disorders and FDR thresholds respectively. The third column (m_0) is the mean gene size of genes with FDRs less than the FDR threshold on the second column. m_1 is a vector of gene-size means obtained from a permutation process.

Gene set	GN0	GN0	P value	FDR
'constrained'	1003	939	3.33E-06	0.007563308
'pLI09'	3488	3241	1.00E-05	0.010084411
'rbfox2'	3068	2895	1.33E-05	0.010084411
'GGGAGGRR_V$S_MAZ_Q6'	2274	2114	3.50E-05	0.015882921
'ACAGGGT,MIR-10A,MIR-10B'	123	116	3.00E-05	0.015882921
'chd8.human_brain'	2798	2601	5.00E-05	0.018908144
'MP:0005179'	208	199	6.50E-05	0.02106918
'rbfox13'	3445	3230	0.000169998	0.048215768

Table S20: Enrichment of gene sets from different databases with SCZ genes from `extTADA` results. These p values were obtained by 10,000,000 simulations, and then adjusted by using the method of Benjamini and Hochberg (1995).

Table S21: The p values of enrichment tests for known gene sets in SCZ, DD, ID, ASD and EPI (See LongSupTables.xlsx Download).

Table S22: The p values of enrichment tests for all gene sets in SCZ, DD, ID, ASD and EPI (See LongSupTables.xlsx Download).

Table S23: Community memberships of the GeNets InWeb PPI 288 NDD genes network.

Table S24: Enrichment results of GeNets. These are enrichment results of 6 communities obtained from GeNets.

References

À. Bayés, L. N. van de Lagemaat, M. O. Collins, M. D. Croning, I. R. Whittle, J. S. Choudhary, and S. G. Grant. Characterization of the proteome, diseases and evolution of the human postsynaptic density. *Nature neuroscience*, 14(1): 19–21, 2011.

Y. Benjamini and Y. Hochberg. Controlling the false discovery rate: a practical and powerful approach to multiple testing. *Journal of the royal statistical society. Series B (Methodological)*, pages 289–300, 1995.

B. Carpenter, A. Gelman, M. Hoffman, D. Lee, B. Goodrich, M. Betancourt, M. A. Brubaker, J. Guo, P. Li, and A. Riddell. Stan: a probabilistic programming language. *Journal of Statistical Software*, 2015.

J. Cotney, R. A. Muhle, S. J. Sanders, L. Liu, A. J. Willsey, W. Niu, W. Liu, L. Klei, J. Lei, J. Yin, et al. The autism-associated chromatin modifier chd8 regulates other autism risk genes during human neurodevelopment. *Nature communications*, 6, 2015.

J. C. Darnell, S. J. Van Driesche, C. Zhang, K. Y. S. Hung, A. Mele, C. E. Fraser, E. F. Stone, C. Chen, J. J. Fak, S. W. Chi, et al. Fmrp stalls ribosomal translocation on mrnas linked to synaptic function and autism. *Cell*, 146(2): 247–261, 2011.

J. De Ligt, M. H. Willemsen, B. W. van Bon, T. Kleefstra, H. G. Yntema, T. Kroes, A. T. Vulto-van Silfhout, D. A. Koolen, P. de Vries, C. Gilissen, et al. Diagnostic exome sequencing in persons with severe intellectual disability. *New England Journal of Medicine*, 367(20):1921–1929, 2012.

S. De Rubeis, X. He, A. P. Goldberg, C. S. Poultney, K. Samocha, A. E. Cicek, Y. Kou, L. Liu, M. Fromer, S. Walker, et al. Synaptic, transcriptional and chromatin genes disrupted in autism. *Nature*, 515(7526):209–215, 2014.

Deciphering Developmental Disorders Study. Prevalence and architecture of de novo mutations in developmental disorders. *Nature*, 542(7642):433–438, 2017.

EuroEPINOMICS-RES Consortium, Epilepsy Phenome/Genome Project, and Epi4K Consortium. De novo mutations in synaptic transmission genes including dnm1 cause epileptic encephalopathies. *The American Journal of Human Genetics*, 95(4):360–370, 2014.

C. Fraley and A. E. Raftery. Mclust: Software for model-based cluster analysis. *Journal of Classification*, 16(2):297–306, 1999.

M. Fromer, A. J. Pocklington, D. H. Kavanagh, H. J. Williams, S. Dwyer, P. Gormley, L. Georgieva, E. Rees, P. Palta, D. M. Ruderfer, et al. De novo mutations in schizophrenia implicate synaptic networks. *Nature*, 506(7487): 179–184, 2014.

G. Genovese, M. Fromer, E. A. Stahl, D. M. Ruderfer, K. Chambert, M. Landen, J. L. Moran, S. M. Purcell, P. Sklar, P. F. Sullivan, C. M. Hultman, and S. A. McCarroll. Increased burden of ultra-rare protein-altering variants among 4,877 individuals with schizophrenia. *Nat Neurosci*, advance online publication:–, 10 2016. URL http://dx.doi.org/10.1038/nn.4402.

S. L. Girard, J. Gauthier, A. Noreau, L. Xiong, S. Zhou, L. Jouan, A. Dionne-Laporte, D. Spiegelman, E. Henrion, O. Diallo, et al. Increased exonic de novo mutation rate in individuals with schizophrenia. *Nature genetics*, 43(9): 860–863, 2011.

M. Guipponi, F. A. Santoni, V. Setola, C. Gehrig, M. Rotharmel, M. Cuenca, O. Guillin, D. Dikeos, G. Georgantopoulos, G. Papadimitriou, et al. Exome sequencing in 53 sporadic cases of schizophrenia identifies 18 putative candidate genes. *PloS one*, 9(11):e112745, 2014.

S. Gulsuner, T. Walsh, A. C. Watts, M. K. Lee, A. M. Thornton, S. Casadei, C. Rippey, H. Shahin, V. L. Nimgaonkar, R. C. Go, et al. Spatial and temporal mapping of de novo mutations in schizophrenia to a fetal prefrontal cortical network. *Cell*, 154(3):518–529, 2013.

F. F. Hamdan, M. Srour, J.-M. Capo-Chichi, H. Daoud, C. Nassif, L. Patry, C. Massicotte, A. Ambalavanan, D. Spiegelman, O. Diallo, et al. De novo mutations in moderate or severe intellectual disability. *PLoS Genet*, 10(10): e1004772, 2014.

X. He, S. J. Sanders, L. Liu, S. De Rubeis, E. T. Lim, J. S. Sutcliffe, G. D. Schellenberg, R. A. Gibbs, M. J. Daly, J. D. Buxbaum, et al. Integrated model of de novo and inherited genetic variants yields greater power to identify risk genes. *PLoS Genet*, 9(8):e1003671, 2013.

I. Iossifov, M. Ronemus, D. Levy, Z. Wang, I. Hakker, J. Rosenbaum, B. Yamrom, Y.-h. Lee, G. Narzisi, A. Leotta, et al. De novo gene disruptions in children on the autistic spectrum. *Neuron*, 74(2):285–299, 2012.

X. Ji, R. L. Kember, C. D. Brown, and M. Buan. Increased burden of deleterious variants in essential genes in autism spectrum disorder. *Proceedings of the National Academy of Sciences*, 2016. doi: 10.1073/pnas.1613195113.

M. R. Johnson, K. Shkura, S. R. Langley, A. Delahaye-Duriez, P. Srivastava, W. D. Hill, O. J. Rackham, G. Davies, S. E. Harris, A. Moreno-Moral, et al. Systems genetics identifies a convergent gene network for cognition and neurodevelopmental disease. *Nature neuroscience*, 19(2):223–232, 2016.

G. Kirov, A. Pocklington, P. Holmans, D. Ivanov, M. Ikeda, D. Ruderfer, J. Moran, K. Chambert, D. Toncheva, L. Georgieva, et al. De novo cnv analysis implicates specific abnormalities of postsynaptic signalling complexes in the pathogenesis of schizophrenia. *Molecular psychiatry*, 17(2):142–153, 2012.

M. Lek, K. Karczewski, E. Minikel, K. Samocha, E. Banks, T. Fennell, A. O'Donnell-Luria, J. Ware, A. Hill, B. Cummings, et al. Analysis of protein-coding genetic variation in 60,706 humans. *bioRxiv*, page 030338, 2015.

S. H. Lelieveld, M. R. Reijnders, R. Pfundt, H. G. Yntema, E.-J. Kamsteeg, P. de Vries, B. B. de Vries, M. H. Willemsen, T. Kleefstra, K. Löhner, et al. Meta-analysis of 2,104 trios provides support for 10 new genes for intellectual disability. *Nature Neuroscience*, 19(9):1194–1196, 2016.

M. Lin, A. Hrabovsky, E. Pedrosa, T. Wang, D. Zheng, and H. M. Lachman. Allele-biased expression in differentiating human neurons: implications for neuropsychiatric disorders. *PLoS One*, 7(8):e44017, 2012.

K. Lindblad-Toh, M. Garber, O. Zuk, M. F. Lin, B. J. Parker, S. Washietl, P. Kheradpour, J. Ernst, G. Jordan, E. Mauceli, et al. A high-resolution map of human evolutionary constraint using 29 mammals. *Nature*, 478(7370):476–482, 2011.

C. Loader. Locfit: Local regression, likelihood and density estimation. *R package version*, 1, 2007.

S. E. McCarthy, J. Gillis, M. Kramer, J. Lihm, S. Yoon, Y. Berstein, M. Mistry, P. Pavlidis, R. Solomon, E. Ghiban, et al. De novo mutations in schizophrenia implicate chromatin remodeling and support a genetic overlap with autism and intellectual disability. *Molecular psychiatry*, 19(6):652, 2014.

B. J. ORoak, L. Vives, S. Girirajan, E. Karakoc, N. Krumm, B. P. Coe, R. Levy, A. Ko, C. Lee, J. D. Smith, et al. Sporadic autism exomes reveal a highly interconnected protein network of de novo mutations. *Nature*, 485(7397):246–250, 2012.

M. Pirooznia, T. Wang, D. Avramopoulos, D. Valle, G. Thomas, R. L. Huganir, F. S. Goes, J. B. Potash, and P. P. Zandi. Synaptomedb: an ontology-based knowledgebase for synaptic genes. *Bioinformatics*, 28(6):897–899, 2012.

R Core Team. *R: A Language and Environment for Statistical Computing*. R Foundation for Statistical Computing, Vienna, Austria, 2016. URL https://www.R-project.org/.

A. Rauch, D. Wieczorek, E. Graf, T. Wieland, S. Endele, T. Schwarzmayr, B. Albrecht, D. Bartholdi, J. Beygo, N. Di Donato, et al. Range of genetic mutations associated with severe non-syndromic sporadic intellectual disability: an exome sequencing study. *The Lancet*, 380(9854):1674–1682, 2012.

E. B. Robinson, B. M. Neale, and S. E. Hyman. Genetic research in autism spectrum disorders. *Current opinion in pediatrics*, 27(6):685, 2015.

K. E. Samocha, E. B. Robinson, S. J. Sanders, C. Stevens, A. Sabo, L. M. McGrath, J. A. Kosmicki, K. Rehnström, S. Mallick, A. Kirby, et al. A framework for the interpretation of de novo mutation in human disease. *Nature genetics*, 46(9):944–950, 2014.

S. J. Sanders, M. T. Murtha, A. R. Gupta, J. D. Murdoch, M. J. Raubeson, A. J. Willsey, A. G. Ercan-Sencicek, N. M. DiLullo, N. N. Parikshak, J. L. Stein, et al. De novo mutations revealed by whole-exome sequencing are strongly associated with autism. *Nature*, 485(7397):237–241, 2012.

T. Singh, M. I. Kurki, D. Curtis, S. M. Purcell, L. Crooks, J. McRae, J. Suvisaari, H. Chheda, D. Blackwood, G. Breen, et al. Rare loss-of-function variants in setd1a are associated with schizophrenia and developmental disorders. *Nature neuroscience*, 2016.

C. Spearman. The proof and measurement of association between two things. *The American journal of psychology*, 15(1):72–101, 1904.

T. N. Turner, Q. Yi, N. Krumm, J. Huddleston, K. Hoekzema, H. A. Stessman, A.-L. Doebley, R. A. Bernier, D. A. Nickerson, and E. E. Eichler. denovo-db: a compendium of human de novo variants. *Nucleic Acids Research*, page gkw865, 2016.

J. L. Wagnon, M. Briese, W. Sun, C. L. Mahaffey, T. Curk, G. Rot, J. Ule, and W. N. Frankel. Celf4 regulates translation and local abundance of a vast set of mrnas, including genes associated with regulation of synaptic function. *PLoS Genet*, 8(11):e1003067, 2012.

S. M. Weyn-Vanhentenryck, A. Mele, Q. Yan, S. Sun, N. Farny, Z. Zhang, C. Xue, M. Herre, P. A. Silver, M. Q. Zhang, et al. Hits-clip and integrative modeling define the rbfox splicing-regulatory network linked to brain development and autism. *Cell reports*, 6(6):1139–1152, 2014.

B. Xu, I. Ionita-Laza, J. L. Roos, B. Boone, S. Woodrick, Y. Sun, S. Levy, J. A. Gogos, and M. Karayiorgou. De novo gene mutations highlight patterns of genetic and neural complexity in schizophrenia. *Nature genetics*, 44(12): 1365–1369, 2012.
